# Metabolome contribution to sex differences in the link between alcohol consumption and type 2 diabetes: a prospective analysis in the Hispanic Community Health Study/Study of Latinos

**DOI:** 10.1016/j.ajcnut.2026.101203

**Published:** 2026-01-21

**Authors:** Brian Wang, Kai Luo, Wenyan Ma, Yanbo Zhang, Christina Cordero, Amber Pirzada, Martha Daviglus, Krista M Perreira, Bing Yu, Eric Boerwinkle, Robert C Kaplan, Qibin Qi

**Affiliations:** 1 Department of Epidemiology and Population Health, Albert Einstein College of Medicine, Bronx, NY, United States; 2 Department of Psychology, University of Miami, Miami, FL, United States; 3 Institute for Minority Health Research, University of Illinois at Chicago, Chicago, IL, United States; 4 Department of Preventive Medicine, University of Illinois at Chicago, Chicago, IL, United States; 5 Department of Social Medicine, University of North Carolina School of Medicine, Chapel Hill, NC, United States; 6 Department of Epidemiology, School of Public Health, UTHealth Houston, Houston, TX, United States

**Keywords:** alcohol consumption, sex difference, metabolome, type 2 diabetes, insulin resistance

## Abstract

**Background:**

Light-to-moderate alcohol consumption has been linked to improved insulin resistance and lower type 2 diabetes (T2D) risk predominantly in females but not males. Potential mechanisms underlying this sex difference remain unclear.

**Objectives:**

This study evaluated associations of sex-specific alcohol-associated metabolomic signatures (AMSs) with insulin resistance and T2D risk in United States Hispanic/Latino adults.

**Methods:**

We analyzed serum metabolome data in the Hispanic Community Health Study/Study of Latinos, a prospective, multicenter, community-based study of Hispanics/Latinos, aged 18 to 74 y old, enrolled from 4 United States metropolitan areas between 2008 and 2011. Sex-specific AMSs were developed using an elastic net to identify serum metabolites uniquely associated with alcohol consumption in females (*n* = 2747) and males (*n* = 1737) without diabetes at baseline, respectively, excluding heavy drinkers. Poisson regression was used to examine the cross-sectional associations of AMSs with insulin resistance (homeostasis model assessment of insulin resistance ≥2.5) and the prospective associations of AMSs with T2D risk in females (*n* = 2265) and males (*n* = 1290) over ∼6 y, adjusting for demographic, socioeconomic, and behavioral factors.

**Results:**

We identified 40 and 54 metabolites uniquely associated with light-to-moderate alcohol consumption in females and males, respectively. Cross-sectionally, female-specific AMS (FAMS) was inversely associated with insulin resistance and various T2D-related metabolic traits in females, whereas male-specific AMS was positively associated with insulin resistance and metabolic traits in males. Prospectively, females in the highest quartile of FAMS had ∼82% (95% confidence interval: 70%, 89%) lower T2D risk compared with those in the lowest quartile. The favorable association between alcohol consumption and risk of T2D was attenuated after adjusting for FAMS. In males, there was no statistically significant association between male-specific AMS and T2D risk.

**Conclusions:**

Our results suggested distinct blood metabolomic signatures associated with alcohol consumption in females and males, which might contribute to sex differences in the relationship between alcohol consumption and T2D.

## Introduction

Prevalence of type 2 diabetes (T2D) has increased significantly in the last 2 decades in the United States, affecting >14% of adults [1]. Sex differences in T2D have been observed, as males show a higher prevalence of T2D than females [2], whereas females appear to bear a greater risk factor burden at the time of their T2D diagnosis [3] and have a higher vascular risk associated with diabetes compared with males [4]. Insulin resistance, characterized by the impaired ability of tissues to respond to insulin and use glucose from the blood, is a key factor responsible for the development of T2D [5,6]. Effectively addressing insulin resistance may reduce T2D and its complications, such as cardiovascular disease, in both males and females [7]. Epidemiologic studies have suggested that light-to-moderate alcohol consumption is associated with a lower risk of T2D, and this protective effect is more pronounced in females than in males [8,9]. This may be explained by female-specific improvements in insulin resistance associated with alcohol consumption [10,11]. A systematic meta-analysis of intervention studies demonstrated that alcohol consumption can reduce fasting insulin concentrations and improve insulin resistance in females, whereas no such effect was observed in males [12]. However, the observed sex differences in the associations of alcohol consumption with insulin resistance and risk of T2D and the underlying biological mechanisms are not well-understood.

The blood metabolome, a collection of small molecules generated through key metabolic processes, has emerged as a valuable tool for uncovering potential biological mechanisms linking diet and lifestyle factors, such as alcohol consumption, to human health and diseases [13]. Previous studies have shown that alcohol consumption was associated with significant changes in circulating metabolites. For example, higher alcohol consumption has been associated with higher concentrations of steroid metabolites, phosphatidylcholine diacyls, and fatty acids, and lower concentrations of phosphatidylcholine acyl-alkyls and sphingomyelins. Higher alcohol consumption has also been associated with altered circulating amino acid concentrations, including higher concentrations of tyrosine and alanine, and lower concentrations of glutamine [[14], [15], [16], [17]]. Many of these alcohol consumption-associated circulating metabolites have been linked to insulin resistance and T2D [18,19]. However, the interrelationship among alcohol consumption, circulating metabolites, and insulin resistance, as well as the risk of T2D, has not been well-studied. Particularly, it remains unclear whether the blood metabolomic profile associated with alcohol consumption may contribute to the observed sex differences in the associations of alcohol consumption with insulin resistance and risk of T2D.

In this study, leveraging serum metabolome data in the Hispanic Community Health Study/Study of Latinos (HCHS/SOL), we aimed to identify potential sex-specific serum metabolomic signatures associated with alcohol consumption and examine whether the identified serum metabolomic signatures may partly explain sex differences in the associations of alcohol consumption with insulin resistance and risk of T2D. The HCHS/SOL is well-suited to address these research questions given its large sample size, prospective study design, varied alcohol consumption patterns of participants, and serum metabolomics data, as well as extensive data on demographic, socioeconomic, behavioral, and clinical factors, including glycemic traits to define insulin resistance and T2D.

## Methods

### Study design and population

**FIGURE 1:**
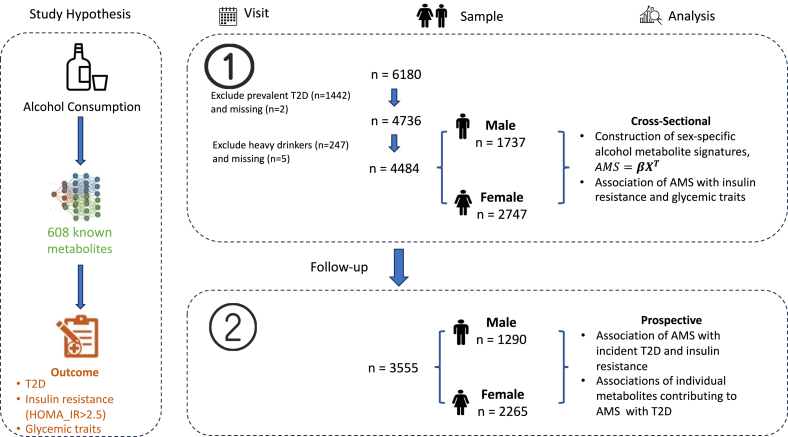
Overview of study design and data analysis. AMS, alcohol-associated metabolomic signature; T2D, type 2 diabetes.

The HCHS/SOL is an ongoing prospective cohort study of United States Hispanic/Latino persons with 16,415 adults aged 18 to 74 y, recruited from 4 United States metropolitan areas (Miami, San Diego, Chicago, and the Bronx area of New York) during 2008 to 2011 [baseline, visit 1] through a multi-stage sampling survey design [20,21]. Interviewer-administered questionnaires were used to collect information on age, sex, annual household income, educational attainment, Hispanic/Latino background, cigarette use history, alcohol consumption, self-reported health, birth nationality, menopause status, ever use of birth control medication, and hormone replacement therapy in the past 12 mo. The alternative healthy eating index-2010 (AHEI-2010) [22] was calculated based on 2 24-h dietary recalls using the National Cancer Institute methodology (https://epi.grants.cancer.gov/diet/usualintakes/), which accounts for within-individual variation across recalls and related covariates (age, sex, field center, Hispanic/Latino background, weekend/weekday, and self-reported difference between intake amount on the survey day and usual intake amount) [23]. Detailed information on sociodemographic, behaviors/lifestyle, medication use, and disease history was collected at baseline and updated during visit 2 (2014‒2017). Anthropometric and biochemical assessments were performed following standardized methods and protocols. In this study, we included ≤6180 participants with serum metabolomic data at baseline. After excluding participants with prevalent diabetes (*n* = 1442), heavy drinkers (*n* = 247), and missing information on diabetes or alcohol consumption (*n* = 7) at baseline, a total of 4484 participants (*n* = 1737 males and 2747 females) were included in the current analyses. We excluded participants with diabetes because diabetes and its treatments (e.g., metformin use) can substantially alter the blood metabolomic profile. Moreover, diabetes may influence alcohol consumption and dietary/lifestyle behaviors, potentially confounding the associations under investigation. We excluded heavy drinkers because, in contrast to light-to-moderate drinkers, heavy alcohol consumption was reported to be associated with an increased risk of T2D [8,9]. All participants provided written informed consent, and the study was approved by the institutional review boards of each participating institute. An overview of the study design and statistical analyses of the present study is shown in Figure 1.

### Alcohol consumption assessment

Alcohol consumption was assessed at baseline by a questionnaire adapted for use in United States Hispanic/Latino adults [24]. After excluding alcohol heavy drinkers (≥15 per week for males and ≥8 drinks per week for females [25]) at baseline, 2 alcohol consumption variables were used in our analyses. Baseline alcohol consumption status was defined as a binary variable of current drinkers and nondrinkers (including former drinkers and never drinkers). Alcohol consumption level was defined as a variable of 5 levels: never drinker, former drinker, current drinker with ≤2 drinks per week, current drinker with 3 to 5 drinks per week, and current drinker with ≥6 drinks per week.

### Insulin resistance, T2D and cardiometabolic traits

Diabetes was defined if participants met 1 of the following criteria as described previously [26]: *1*) fasting glucose ≥7.0 mmol/L (126 mg/dL) if fasting time >8 h or ≥11.1 mmol/L (200 mg/dL) if fasting time ≤8 h; *2*) 2-hour postprandial glucose (PPG) ≥11.1 mmol/L (200 mg/dL); *3*) hemoglobin A1c (HbA1c) ≥6.5%; *4*) current use of antidiabetic medications, or self-reported physician diagnosed T2D. Participants without diabetes at baseline who met the above criteria at the visit 2 follow-up examination were defined as incident T2D cases. Insulin resistance was defined as a homeostasis model assessment of insulin resistance (HOMA-IR) score of ≥2.5 [[27], [28], [29]]. Continuous T2D-related metabolic traits, including body mass index (BMI) (in kg/m^2^), fasting insulin, fasting plasma glucose (FPG), HbA1c, PPG, HOMA-IR $\left[\frac{\text{fasting}\;\text{glucose}\;\left(\frac{\text{mg}}{\text{dL}}\right)\times \text{fasting}\;\text{insulin}\;\left(\frac{\text{mU}}{L}\right)}{405}\right]$, high-density lipoprotein (HDL) cholesterol, and triglycerides (TG), were measured at baseline using standard methods [26,30].

### Blood metabolome profiling

The serum metabolome was profiled using the DiscoveryHD4 platform at Metabolon. Detailed methods for mass spectrometry (MS) analysis, metabolite identification, and quality control (QC) have been previously reported [31]. In brief, serum samples were extracted with methanol and subjected to non-targeted mass MS analysis using UPLC-MS/MS. Metabolites were identified by automated comparison of the ion features in the experimental samples to a reference library of chemical standard entries that included retention time, molecular weight (*m/z*), preferred adducts, and in-source fragments, as well as associated MS spectra, and curated by visual inspection for QC using software developed at Metabolon. Pooled QC plasma replicates from study samples were used to determine endogenous biochemical variability, with a representative SD of 10% across all metabolites. A total of 608 known metabolites detected in >90% of participants at baseline were included in the association analyses. Values of metabolites below detection were imputed by the half of the minimum value. Rank-based inverse normal transformation was performed to improve the normality and alleviate the impacts of extreme values prior to association analyses.

### Statistical analysis

The statistical software R (version 4.0.2; The R Foundation for Statistical Computing) was used for all analyses. Clinical characteristics of study participants were summarized by sex. Categorical variables were presented as frequencies, and continuous variables were presented as medians and IQR.

Linear regression models were first used to compare levels of individual metabolites among current, former, and never alcohol drinkers in females and males, respectively, at baseline. Models were adjusted for age, study center, birth nationality, smoking status, income, education, AHEI-2010, Hispanic/Latino background, and metabolomic profiling batch in males and females, and additionally, menopause status, ever use of birth control medication, and hormone replacement therapy in females (Table 1). For variables with missing data [AHEI-2010 (<1%), income (6.3%), menopausal status (15.5% in females), ever use of birth control medication (10.9% in females), and hormone replacement therapy (2% in females)], the missing values were imputed by sex using the mice package in R.

**TABLE 1:** Characteristics of study participants at baseline.

	Female	Male
*n* (%)	2747 (61.3)	1737 (38.7)
Age groups (%)		
18‒29	221 (8)	189 (11)
30‒39	270 (10)	214 (12)
40‒49	453 (16)	297 (17)
50‒59	1022 (37)	585 (34)
60‒69	613 (22)	342 (20)
71‒74	168 (6)	110 (6)
United States born (%)	427 (16)	329 (19)
Current smoker (%)	429 (16)	457 (26)
Income (%)		
<$30,000	1840 (71)	1007 (60)
≥$30,000	735 (29)	662 (40)
Education (%)		
Less than high school	928 (34)	575 (33)
High school or equivalent	677 (25)	488 (28)
Greater than high school or equivalent	1138(41)	672 (39)
Study center (%)		
Miami	787 (29)	528 (30)
San Diego	618 (22)	352 (20)
Chicago	601 (22)	447 (26)
Bronx	741 (27)	410 (24)
Hispanic backgrounds (%)		
Central American	306 (11)	171 (10)
Cuban	453 (17)	358 (21)
Dominican	334 (12)	157 (9)
Mexican	948 (35)	558 (32)
Puerto Rican	418 (15)	308 (18)
South American	209 (8)	133 (8)
Other	75 (3)	49 (3)
Alcohol consumption (%)		
Never drinker	752 (27)	177 (10)
Past drinker	899 (33)	528 (30)
≤2 drinks a wk	825(30)	434 (25)
3‒5 drinks a wk	191 (7)	221 (13)
≥6 drinks a wk	80 (3)	377 (22)
Menopause (%)		
No	1334 (57.4)	—
Yes	988(42.6)	—
Ever use birth control medication (%)		
No	930 (38)	—
Yes	1518 (62)	—
Hormone replacement therapy (%)		
No	2596 (96)	—
Yes	100 (4)	—
AHEI-2010	47.5 (42.4‒52.7)	48.8 (43.8‒54.6)
Insulin Resistance (HOMA-IR 2.5, %)	1191 (43)	800 (46)
BMI (kg/m^2^)	28.8 (25.6‒33.1)	28.1 (25.2‒31.2)
Fasting glucose (mg/dL)	92 (87‒97)	96 (90‒101)
HbA1c (mmol mol^‒1^)	37 (34‒39.8)	37 (34‒39)
Fasting insulin (mIU/L)	10.0 (6.8‒14.8)	9.8 (6.0‒15.2)
HOMA-IR	2.3 (1.5‒3.5)	2.4 (1.4‒3.7)
PPG (mg/dL)	118 (99‒140)	109 (88‒132)
HDL cholesterol (mg/dL)	52 (45‒61)	44 (38‒51)
TG (mg/dL)	104 (75‒147)	124 (83‒183)

Values are denoted as median (IQR1, IQR3).

Abbreviations: AHEI-2010, alternative healthy eating index-2010; BMI, body mass index; HbA1c, hemoglobin A1c; HDL, High-density lipoprotein; HOMA-IR, homeostasis model assessment of insulin resistance; PPG, 2-hour postprandial; TG, triglycerides.

Elastic-net models (using the glmnet R package with 10-fold cross-validation) were then used to select metabolites associated with alcohol consumption status (i.e., current drinkers compared with noncurrent drinkers). The respective AUCs were calculated by the receiver operating characteristic curve created from the predicted probabilities for alcohol consumption status produced by the elastic-net models. Linear regression models were used to examine associations of selected metabolites with alcohol consumption status in females and males, after adjustment for covariate described above. The interaction between alcohol consumption and sex was tested by including an interaction term in the model. Sex-specific alcohol-associated metabolomic signatures (*AMS*) were defined as $AMS=\beta X^{T}\text{,}$ representing a linear combination of levels of unique metabolites identified in a specific sex ($X^{T}$) weighted by corresponding coefficients (β) derived from the elastic-net model.

In the cross-sectional analysis, we aimed to examine the associations of sex-specific AMSs with insulin resistance and metabolic traits at baseline. Poisson regression with robust variance was applied for insulin resistance, and rank-based robust regression models were used for continuous metabolic traits, including HOMA-IR, BMI, fasting insulin, FPG, PPG, HbA1c, HDL cholesterol, and TG. In these models, AMSs were categorized into 4 levels [Q1 (reference), Q2, Q3, and Q4] based on quartiles in females and males, respectively, with adjustment for the same covariates described above. Prevalence ratios (PRs), 95% confidence intervals (CIs), and *P*-trend across quartiles were calculated in Poisson regression by treating sex-specific AMS quartile as an ordinal variable assigned values of 0 (Q1, reference), 1 (Q2), 2 (Q3), and 3 (Q4). Similar regression models were also applied to examine associations of alcohol consumption levels with insulin resistance and metabolite traits, and the regression coefficients from models with and without adjustment for sex-specific AMSs were compared. PRs, 95% CIs and *P*-trend across alcohol consumption levels were calculated in Poisson regression by treating alcohol consumption levels as an ordinal variable assigned values of 0 (never, reference), 1 (former), 2 (≤2 drinks per week), 3 (3‒5 drinks per week), and 4 (5‒6 drinks per week for females and 5‒14 drinks per week for males).

In the prospective analysis, we aimed to examine whether baseline sex-specific AMSs were associated with incident T2D and incident insulin resistance at visit 2. Poisson regression models with a robust variance estimator were applied, adjusting for the aforementioned covariates. Similar Poisson regression models were applied to examine associations of alcohol consumption levels with risk of T2D, and regression coefficients from models with and without adjustment for sex-specific AMSs were compared. Relative risks (RRs), 95% CIs, and *P* for trend across sex-specific AMS quartiles and alcohol consumption levels were calculated in Poisson regression by treating sex-specific AMS quartile and alcohol consumption levels as an ordinal variable, respectively, as described above. Prospective associations between individual AMS-related metabolites and incident T2D were also assessed using Poisson regression models. Finally, the correlation between the estimated regression coefficients for cross-sectional associations between metabolites and alcohol consumption and prospective associations between metabolites and incident T2D was evaluated using Pearson’s correlation analysis.

In the analyses above, sex-specific AMSs were derived using cross-validated elastic-net regression based on a large number of metabolites, and their associations with alcohol consumption and outcome variables were assessed in the same dataset. Because the potential overfitting in the elastic net could bias the estimated associations of AMSs with alcohol consumption and outcome variables, we conducted a sensitivity analysis to assess the robustness of our findings. Specifically, we randomly and evenly split the dataset into a training set for deriving the AMSs and a validation set for testing their associations with alcohol consumption and outcome variables, applying the same analytic approaches described above.

## Results

### Characteristics of study participants

Participant characteristics are shown in Table 1 and Supplemental Table 1. Among 4484 participants free of diabetes at baseline in our analysis, 2747 (61.3%) were females, and 1737 (38.7%) were males. Compared with males, females were slightly older, had lower income, a slightly higher BMI, and better metabolic traits (lower FPG and lower TG). A greater proportion of females were never drinkers (27%) and nonsmokers (84%) compared with males (10% and 74%, respectively).

### Metabolomic signatures of alcohol consumption

We compared levels of individual metabolites among current, former, and never drinkers in females and males, respectively, adjusting for the covariates described above. The metabolomic profiles were similar between former and never drinkers, with no significantly differentiated metabolites identified in either females or males (false discovery rate q-value [FDR-q] <0.1). In contrast, significantly differentiated metabolites were identified between current and former drinkers (15 metabolites in females and 30 in males) as well as between current and never drinkers (7 in females and 16 in males, FDR-q <0.1) (Supplemental Figure 1).

**FIGURE 2:**
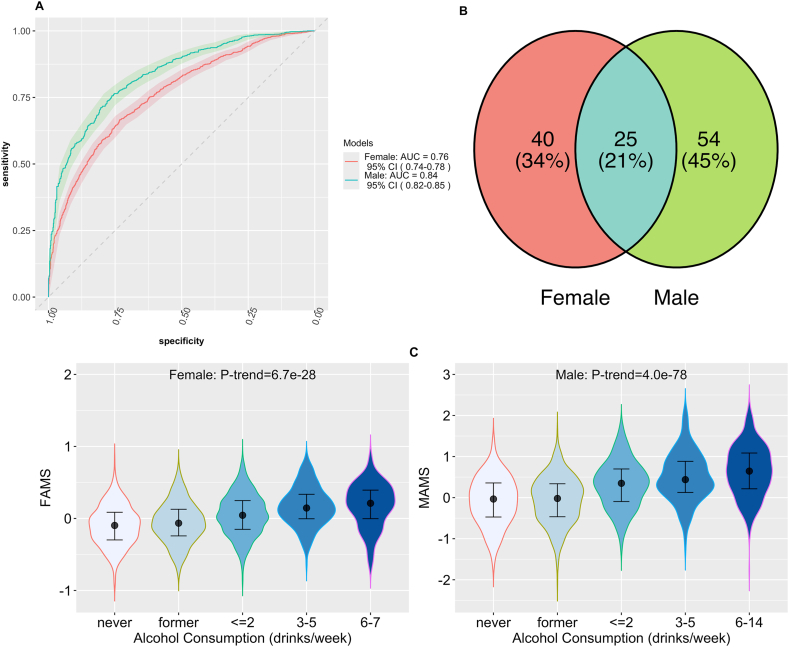
Alcohol consumption and serum metabolite profiles in females and males. (A) Comparison of receiver operating characteristic (ROC) curve of elastic-net models of current alcohol consumption using all 608 known metabolites with 10-fold cross-validation in females (*n* = 2747 red) and males (*n* = 1737 green). The tuning parameter was selected by the minimum criteria. The 95% confidence interval (CI) of ROC (shaded area) was computed with 2000 stratified bootstrap. (B) Venn diagram of identified metabolites associated with alcohol consumption by elastic-net models for females and males. Red: metabolites uniquely identified in females; green: metabolites uniquely identified in males; blue: metabolites identified in both sexes. (C) Violin plot of sex-specific alcohol-associated metabolomic signatures across alcohol consumption levels in females (*FAMS*) and males (*MAMS*). The *P*-trend was obtained by linear regression, in which alcohol consumption level was treated as an ordinal variable assigned values of 0 (never, reference), 1 (former), 2 (≤2 drinks per wk), 3 (3‒5 drinks per wk), and 4 (5‒6 drinks per wk for females and 5‒14 drinks per wk for males), adjusting for age, study center, birth nationality, smoking status, income, education, alternate healthy eating index 2010, Hispanic/Latino background, and metabolomic profiling batch in females and males, and additionally menopause status, ever use of birth control medication, and hormone replacement therapy in females.

We next used elastic-net models to select metabolites that differed between current drinkers and noncurrent drinkers (including both former and never drinkers, given their similar metabolomic profiles). In total, 79 metabolites associated with current alcohol consumption were identified in males, yielding an AUC of 0.84 (95% CI: 0.82, 0.85), and 65 metabolites associated with current alcohol consumption were identified in females, yielding an AUC of 0.76 (95% CI: 0.74, 0.78) (Figure 2A). Although 25 of these metabolites were associated with alcohol consumption in both sexes, many of the selected metabolites were unique to each sex, with 40 unique to females and 54 unique to males (Figure 2B).

We further examined the associations between these uniquely selected metabolites and current alcohol consumption in linear regression models after adjusting for covariates described above. Significant interactions between sex and alcohol consumption (*P*-interaction < 0.05) were observed for 29 metabolites, including 6 and 23 metabolites uniquely selected in females and males, respectively (Supplemental Figure 2 and Supplemental Table 2).

Based on these uniquely selected metabolites associated with alcohol consumption in males (54 metabolites) and females (40 metabolites), we calculated sex-specific *AMSs* and assessed their associations with alcohol consumption levels. We found a highly significant linear association between the sex-specific AMS and alcohol consumption levels in females (P-trend < 0.0001) and males (P-trend < 0.0001), respectively (Figure 2C).

### Cross-sectional associations of metabolomic signatures with insulin resistance and metabolic traits

**FIGURE 3:**
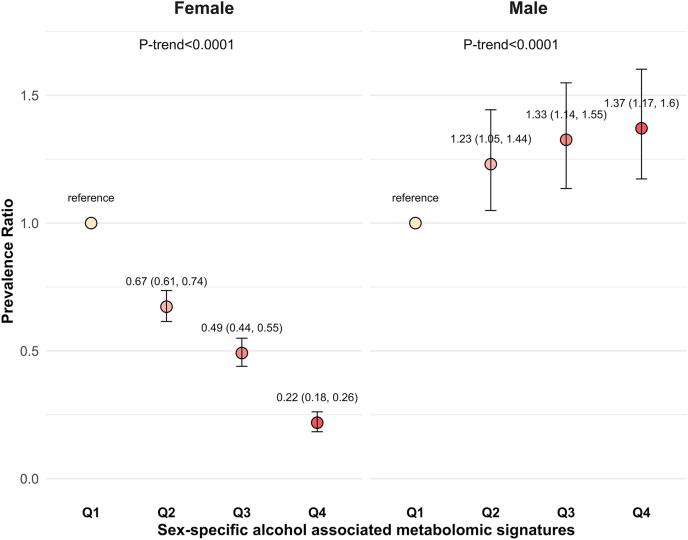
Cross-sectional associations between sex-specific alcohol-associated metabolomic signatures and insulin resistance in females and males at baseline. Females (*n* = 2747) and males (*n* = 1737) were classified into 4 sex-specific alcohol-associated metabolome signatures (AMSs) quartiles [Q1 (reference), Q2, Q3, Q4]. Prevalence ratios (PRs) and 95% confidence intervals (CIs) were estimated using Poisson regression with robust variance, adjusting for age, study center, birth nationality, smoking status, income, education, alternate healthy eating index 2010, Hispanic/Latino background, and metabolomic profiling batch in both sexes and additionally menopause status, ever use of birth control medication, and hormone replacement therapy in females. The *P*-trend was calculated in Poisson regression by treating sex-specific AMS quartile as an ordinal variable assigned values of 0 (Q1, reference), 1 (Q2), 2 (Q3), and 3 (Q4).

In females, female-specific *AMS* (*FAMS*) was negatively associated with insulin resistance [PR (95% CI): 0.67 (0.61, 0.74) for Q2 compared with Q1; 0.49 (0.44, 0.55) for Q3 compared with Q1; 0.22 (0.18, 0.26) for Q4 compared with Q1; *P*-trend < 0.0001]. In contrast, in males, male-specific AMS (*MAMS*) was positively associated with insulin resistance [PR (95% CI): 1.23 (1.05, 1.44) for Q2 compared with Q1; 1.33 (1.14, 1.55) for Q3 compared with Q1; 1.37 (1.17, 1.60) for Q4 compared with Q1; *P*-trend < 0.0001] (Figure 3). There was a negative association between alcohol consumption levels and insulin resistance in females (*P*-trend = 0.053) (Supplemental Figure 3). Compared with never drinkers, drinkers with 6 to 7 drinks a week had ∼28% lower prevalence of insulin resistance [PR: 0.72; 95% CI: 0.51, 1.01] in females. The negative association between alcohol consumption levels and insulin resistance was abolished after adjusting for *FAMS* (Supplemental Figure 3). On the contrary, there was no significant association between alcohol consumption levels and insulin resistance in males. However, after adjusting for *MAMS*, there was a negative association between alcohol consumption levels and insulin resistance (*P*-trend = 0.015) (Supplemental Figure 3).

Additionally, we examined cross-sectional associations of *FAMS* and *MAMS* with T2D-related metabolic traits at baseline (Supplemental Figure 4A). Consistent with the associations observed for insulin resistance, *FAMS* and *MAMS* showed different associations with these traits between females and males. Overall, *FAMS* was negatively associated with BMI, FPG, PPG, fasting insulin, HbA1c, HOMA-IR, and TG (*P*-trend < 0.05) in females, whereas *MAMS* was positively associated with FPG, fasting insulin, HbA1c, HOMA-IR, and TG (*P*-trend < 0.05) in males. Alcohol consumption levels were negatively associated with BMI, fasting insulin, HbA1c, HOMA-IR, and TG in females (*P*-trend < 0.05), whereas these associations were attenuated or even reversed after adjusting for *FAMS*. On the contrary, alcohol consumption levels were positively associated with FPG in males. After adjusting for *MAMS,* the association between alcohol consumption and FPG was attenuated, while alcohol consumption levels became negatively associated with fasting insulin and HOMA-IR in males (Supplemental Figure 4B).

### Prospective associations of metabolomic signatures with incident T2D

**FIGURE 4:**
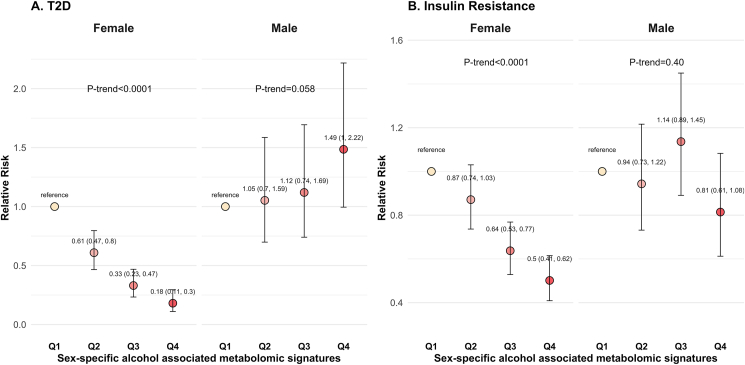
Prospective associations of sex-specific alcohol-associated metabolomic signatures with risk of type 2 diabetes and insulin resistance in females and males. (A) Prospective associations between sex-specific alcohol-associated metabolome signature (AMS) quartiles [Q1 (reference), Q2, Q3, and Q4] at baseline and incident type 2 diabetes over ∼6 y in females (*n* = 2,265) and males (*n* = 1290). Relative risks (RRs) and 95% confidence intervals (CIs) were estimated using Poisson regression, adjusting for age, study center, birth nationality, smoking status, income, education, Alternate Healthy Eating Index 2010 (AHEI-2010), Hispanic/Latino background, and metabolomic profiling batch in both sexes, and additionally menopause status, ever use of birth control medication, and hormone replacement therapy in females. The *P*-trend was calculated by Poisson regression by treating sex-specific AMS quartile as an ordinal variable assigned values of 0 (Q1, reference), 1 (Q2), 2 (Q3), and 3 (Q4). (B) Prospective associations between sex-specific AMS quartiles [Q1 (reference), Q2, Q3, Q4] at baseline and incident insulin resistance over ∼6 y in females (*n* = 978) and males (*n* = 596). RRs and 95% CIs were estimated using Poisson regression, adjusting for age, study center, birth nationality, smoking status, income, education, AHEI-2010, Hispanic/Latino background, and metabolomic profiling batch in both sexes, and additionally, menopause status, ever use of birth control medication, and hormone replacement therapy in females. The *P*-trend was calculated in Poisson regression by treating sex-specific AMS quartile as an ordinal variable assigned values of 0 (Q1, reference), 1 (Q2), 2 (Q3), and 3 (Q4).

We then investigated whether baseline *FAMS* and *MAMS* were associated with incident T2D over ∼6 y in females (*n* = 2265, 255 incident cases) and males (*n* = 1290, 167 incident cases), respectively. A negative association between *FAMS* and risk of T2D was found in females (*P*-trend < 0.0001). When compared with females in the lowest quartile, females in the highest *FAMS* quartile had an 82% lower risk of T2D [RR for Q4 compared with Q1 was 0.18 (95% CI: 0.11, 0.30)] (Figure 4A). In contrast, there was a positive association between *MAMS* and risk of T2D in males (*P*-trend = 0.058). The RR of T2D comparing the highest *MAMS* quartile to the lowest quartile were 1.49 (95% CI: 1.00, 2.22) (Figure 4A).

We found a negative association between alcohol consumption levels at baseline and incident T2D in females, although the trend was not statistically significance (*P*-trend = 0.11). The favorable association between alcohol consumption and incident T2D in females was fully abolished after further adjusting for *FAMS* (*P*-trend = 0.95) (Supplemental Figure 5A). In males, no association between alcohol consumption levels at baseline and incident T2D was observed before (*P*-trend = 0.43) or after further adjusting for *MAMS* (*P*-trend = 0.99) (Supplemental Figure 5A).

Additionally, we investigated whether baseline *FAMS* and *MAMS* were associated with incident insulin resistance in a subset of the participants who were not affected by insulin resistance at baseline (females: *n* = 978, 152 incident cases; males: *n* = 596, 96 incident cases, respectively). Similarly, a negative association between *FAMS* and risk of insulin resistance was found in females (*P*-trend < 0.0001). When compared with females in the lowest quartile, females in the highest *FAMS* quartile had 50% lower risk of insulin resistance [RR for Q4 compared with Q1 was 0.50 (95% CI: 0.41, 0.62)] (Figure 4B). In contrast, there was no significant association between *MAMS* and risk of insulin resistance in males (*P*-trend = 0.40). No significant association was found between alcohol consumption levels and incident insulin resistance before and after adjustment for AMSs in both females and males (Supplemental Figure 5B).

### Individual metabolites in *FAMS* and *MAMS* and incident T2D

Next, we examined associations of individual metabolites contained within *FAMS* and *MAMS* with incident T2D in females and males, respectively. In females, the regression coefficients for associations between individual metabolites and alcohol consumption were inversely correlated with the regression coefficients for the associations between these metabolites and incident T2D (r = ‒0.67, *P* < 0.0001) (Supplemental Figure 6). Among 12 metabolites positively associated with alcohol consumption, 10 metabolites including *1-(1-enyl-palmitoyl)-2-oleoyl-GPC (P-16:0/18:1)* (*P* = 1.7 × 10^‒10^)*, 1-(1-enyl-palmitoyl)-2-linoleoyl-GPC (P-16:0/18:2)* (*P* = 2.7 × 10^‒7^) and hypotaurine (*P* = 1.4 × 10^‒4^), were negatively associated with incident T2D. Among 28 metabolites negatively associated with alcohol consumption, 23 metabolites, including *1-carboxyethylphenylalanine* (*P* = 4.2 × 10^‒14^) and γ*-glutamylisoleucine* (*P* = 3.7 × 10^‒11^), were positively associated with incident T2D.

In contrast, there was a modest positive correlation between the regression coefficients for associations between individual metabolites in *MAMS* and alcohol consumption and the regression coefficients for associations between these metabolites and incident T2D (r = 0.30, *P* = 0.06) (Supplemental Figure 6). Among 17 metabolites negatively associated with alcohol consumption, 6 metabolites (e.g., γ*-glutamylcitrulline*, *P* = 0.009) were negatively associated with incident T2D. Among 24 metabolites positively associated with alcohol consumption, 20 metabolites (e.g., *N-acetylputrescine*, P = 5.5 × 10^‒5^; *2R,3R-dihydroxybutyrate*, P = 4.4 × 10^‒3^) were positively associated with incident T2D.

### Sensitivity analysis

We performed a sensitivity analysis by randomly and evenly splitting into a training dataset for deriving AMSs and a testing dataset for testing associations between AMSs and outcomes in females (*n* = 1374 in the training dataset and *n* = 1373 in the testing dataset) and males (*n* = 869 in the training dataset and *n* = 868 in the testing dataset), respectively. Overall, our sensitivity analysis yielded similar results as observed in our main analysis. In the training dataset, 35 and 25 metabolites were uniquely selected in females and males, respectively, by elastic nets, whereas only 6 were identified in both sexes (Supplemental Figure 7B). In the testing dataset, the elastic-net models achieved an AUC of 0.71 (0.67, 0.74) for males and 0.65 (0.62, 0.68) for females (Supplemental Figure 7A). The derived sex-specific AMSs were highly significantly associated with alcohol consumption levels in males and females in the testing dataset (Supplemental Figure 7C).

In the cross-sectional analysis of the testing dataset at baseline, *FAMS* remained inversely associated with insulin resistance among females (*P*-trend < 0.0001), whereas *MAMS* showed no significant association with insulin resistance among males in the testing dataset (Supplemental Figure 8A). Similarly, *FAMS* and *MAMS* showed different associations with most metabolic traits in females and males, respectively, in the testing dataset (Supplemental Figure 8B).

In prospective analysis in the testing dataset, a negative association between *FAMS* and risk of T2D was found in females (*P*-trend = 0.004). In contrast, there was a positive association between *MAMS* and risk of T2D in males, though it did not reach statistical significance (*P*-trend = 0.17) (Supplemental Figure 9A). Similarly, a negative trend of association between *FAMS* and risk of incident insulin resistance was also found in females, although it did not achieve statistical significance (Supplemental Figure 9B).

## Discussion

In this study of United States Hispanic/Latino adults, we observed sex-based dimorphism in associations between usual alcohol consumption and blood metabolomic profile. In females, a metabolomic signature based on 40 female-specific alcohol-related metabolites showed protective associations with insulin resistance and incident T2D. In males, no significant association was found between a metabolomic signature based on 54 male-specific alcohol-related metabolites and incident T2D. These sex-specific metabolomic profiles associated with alcohol consumption may help explain the previously reported sex differences in the associations of alcohol consumption with insulin resistance and T2D.

Numerous epidemiological studies have investigated the associations between alcohol consumption and serum metabolites in both non-Hispanic White and non-Hispanic Black populations [15,16,[32], [33], [34], [35]]. Alcohol consumption was related to a broad range of metabolite classes, and our findings largely align with previous research. For instance, sex steroids, such as *androstenediol (3*β*,17*β*) monosulfate*, were among the top metabolites positively associated with alcohol consumption in both males and females [17,32,36]. Unlike previous studies, however, our research focused on sex-specific associations between alcohol consumption and circulating metabolites. Overall, more pronounced associations in both the number and the magnitude of alcohol-associated metabolites were observed in males compared with females, whereas unique associations were also observed in females, particularly metabolites within the plasmalogen sub-pathway. The mechanisms behind these sex-specific metabolomic signatures of alcohol consumption remain unclear.

Previous meta-analysis studies suggested that low to moderate alcohol consumption may improve insulin resistance and lower T2D risk, particularly in females [8,12,37]. We observed a similar favorable association among females between alcohol consumption and a variety of metabolic traits as well as incident T2D over ∼6 y. Our study revealed a potential involvement of circulating metabolites in the link between alcohol consumption and insulin resistance and T2D. A negative association between the female-specific metabolomic signature of alcohol consumption and insulin resistance was found in females, and this potentially beneficial association was further confirmed in a prospective analysis of incident insulin resistance and T2D. Among metabolites included in *FAMS*, the top 3 metabolites that were positively associated with alcohol consumption in females—*1-(1-enyl-palmitoyl)-2-oleoyl-GPC (P-16:0/18:1), 1-(1-enyl-palmitoyl)-2-oleoyl-GPC (P-16:0/18:1),* and *hypotaurine*—were associated with a lower risk of T2D in females. Two of them, *1-(1-enyl-palmitoyl)-2-oleoyl-GPC (P-16:0/18:1)* and *1-(1-enyl-palmitoyl)-2-oleoyl-GPC (P-16:0/18:1)*, belong to the plasmalogen sub-pathway that has been associated with a number of diseases, including cardiometabolic diseases [[38], [39], [40]], and might decrease risk of T2D through antioxidative, antiapoptotic, and anti-inflammatory functions [41,42]. Another observed metabolite, *hypotaurine*, a sulfinic acid involved in taurine biosynthesis, plays a role in β-cell function and has antioxidative and anti-inflammatory effects, and it has been linked to insulin resistance and T2D in humans and animals [43]. Metabolites in *FAMS* that were negatively associated with alcohol consumption, such as *1-carboxyethylphenylalanine* and γ*-glutamylisoleucine*, were positively associated with insulin resistance and risk of T2D. *1-carboxyethylphenylalanine*, a phenylalanine derivative, has been reported to exacerbate insulin resistance by stimulating insulin secretion, potentially causing hyperinsulinemia [44,45]. Metabolite γ*-glutamylisoleucine* was also previously linked to unfavorable metabolic health outcomes, including insulin resistance [46]. In contrast, the prospective association between the male-specific metabolomic signature and incident T2D did not achieve statistical significance, although significantly positive cross-sectional associations were observed with insulin resistance and metabolic traits.

Several limitations of the present study should be acknowledged. First, the observational nature of this study may limit its robustness against various confounding factors, making causal inference difficult. Second, because our sex-specific AMSs were derived based on a large number of metabolites in the same dataset where their associations with insulin resistance and T2D were, potential overfitting may bias association estimates, although our sensitivity analyses by splitting the dataset into training and testing subsets yielded similar results. Third, as the study population comprises Hispanic/Latino individuals from diverse backgrounds, generalization of the findings to other populations may require further investigation. Finally, given the limited number of heavy drinkers in the current study and the potential harmful effects of heavy drinking on metabolic health [8,9], we excluded heavy drinkers in our analysis. Future studies with larger sample sizes are needed to examine associations of heavy alcohol consumption with metabolites and T2D.

In summary, this study suggets favorable alterations in the serum metabolome associated with light-to-moderate alcohol consumption in females related to insulin resistance and risk of T2D. Our findings may advance the understanding of biological mechanisms underlying the observed sex differences in the relationships of alcohol consumption with insulin resistance and T2D, potentially facilitating the development of sex-based precision medicine.

## Author contributions

The authors’ responsibilities were as follows – BW: data analysis, study conception, and manuscript preparation; QQ: supervision; KL, WM, YZ, CC, KMP, AP, MD, BY, EB, RCK: critical revision of the manuscript; QQ: guarantor of the work and, as such, had full access to all the data in the study and takes responsibility for the integrity of the data and the accuracy of the data analysis; and all authors: read and approved the final manuscript.

## Data availability

Hispanic community health study/study of Latinos (HCHS/SOL) has established a process for the scientific community to apply for access to participant data and materials, with such requests reviewed by the project’s Steering Committee. These policies are described at https://sites.cscc.unc.edu/hchs/. The corresponding author will accept reasonable requests for data access, which will be referred to the Steering Committee of the HCHS/SOL project. All code regarding the main analyses or data visualization can be made available upon reasonable request to the corresponding author.

## Funding

This work is supported by R01-DK119268, R01DK134672, and R01-DK126698 from the National Institute of Diabetes and Digestive and Kidney Diseases (NIDDK). Other funding sources for this study include R01DK120870 and U01DK140761 from NIDDK; R01HL060712, R01HL168683, and R01HL141824 from the National Heart, Lung, and Blood Institute (NHLBI); R01-MD011389 from the National Institute on Minority Health and Health Disparities; and R01AG085320 from the National Institute on Aging. Additional support was provided by the Life Course Methodology Core of the New York Regional Center for Diabetes Translation Research, funded by the NIDDK (P30DK111022). Support for metabolomics data was provided in part by the JLH Foundation (Austin, TX) and by R01HL141824 from the NHLBI. The Hispanic Community Health Study/Study of Latinos (HCHS/SOL) was supported by contracts from the NHLBI to the University of North Carolina (N01-HC65233), University of Miami (N01-HC65234), Albert Einstein College of Medicine (N01-HC65235), the University of Illinois at Chicago (HHSN268201300003I), Northwestern University (N01-HC65236), and San Diego State University (N01-HC65237). The following Institutes/Centers/Offices contributed to the HCHS/SOL through a transfer of funds to the NHLBI: National Center on Minority Health and Health Disparities, the National Institute on Deafness and Other Communications Disorders, the National Institute of Dental and Craniofacial Research, the NIDDK, the National Institute of Neurologic Disorders and Stroke, and the Office of Dietary Supplements. KL was supported by an American Heart Association postdoctoral fellowship award (23POST1020455).

## Conflict of interest

The authors report no conflicts of interest.
