# Supplementary material for: Metabolome contribution to sex differences in the link between alcohol consumption and type 2 diabetes: a prospective analysis in the Hispanic Community Health Study/Study of Latinos
Source: Am J Clin Nutr. 2026 Jan 21;123(3):101203. doi: 10.1016/j.ajcnut.2026.101203 (PMC12975372; doi:10.1016/j.ajcnut.2026.101203)
Supplement: Multimedia component 1 [file mmc1.docx]

**­­Metabolome contributions to sex difference in the link between alcohol consumption and type 2 diabetes: a prospective analysis in the Hispanic Community Health Study/Study of Latinos (HCHS/SOL)**

Brian Wang

**Supplemental Figures and Tables**

**
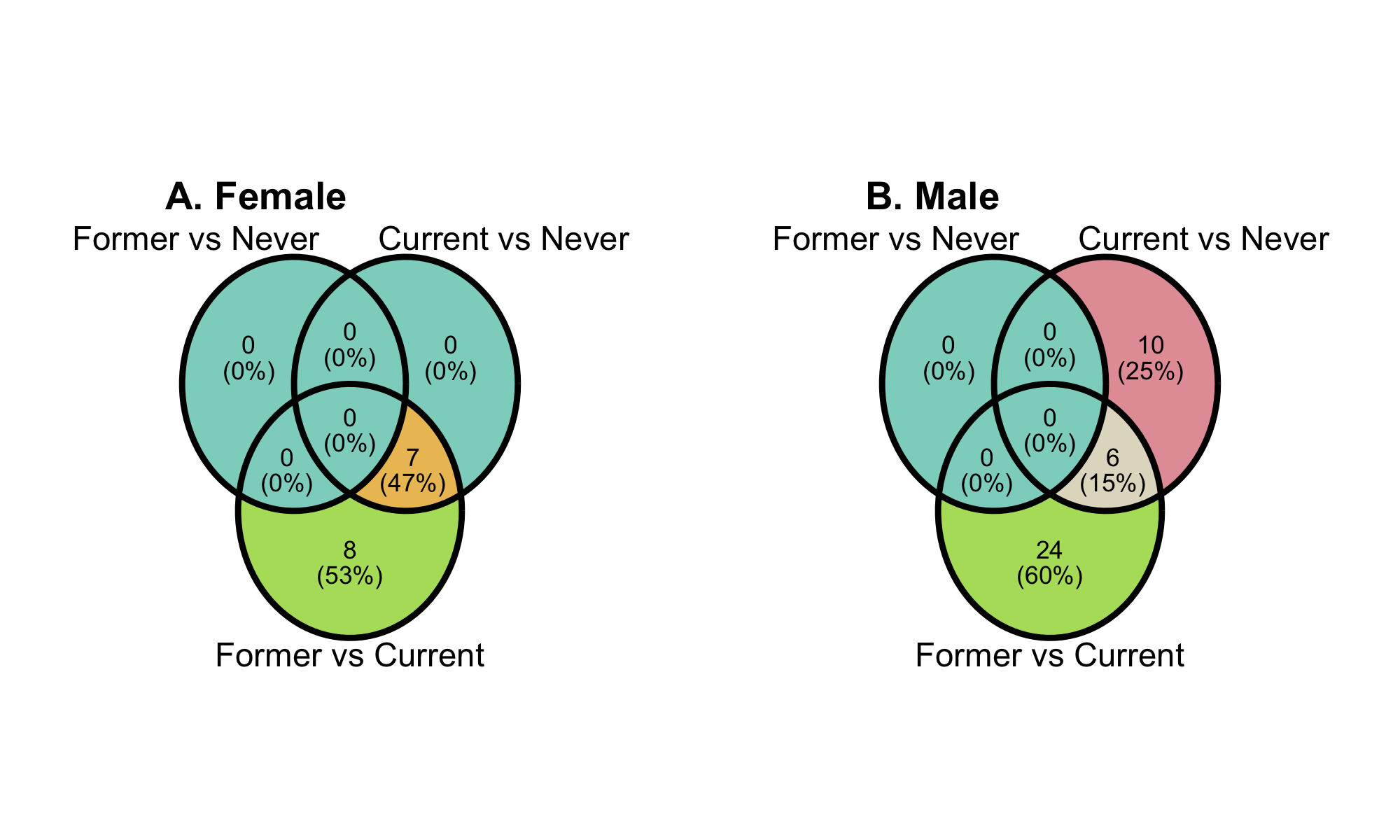
**

**Supplement Figure 1**: Venn diagram of identified differentiated metabolites among current, former, and never smokers by regression models for females and males (FDR-q = 0.1). The associations were adjusted for age, study center, birth nationality, smoking status, income, education, Alternate Healthy Eating Index 2010 (AHEI-2010), Hispanic/Latino background, and metabolomic profiling batch in both sexes. In females, menopause status, ever use of birth control medication, and hormone replacement therapy were additionally adjusted.

**
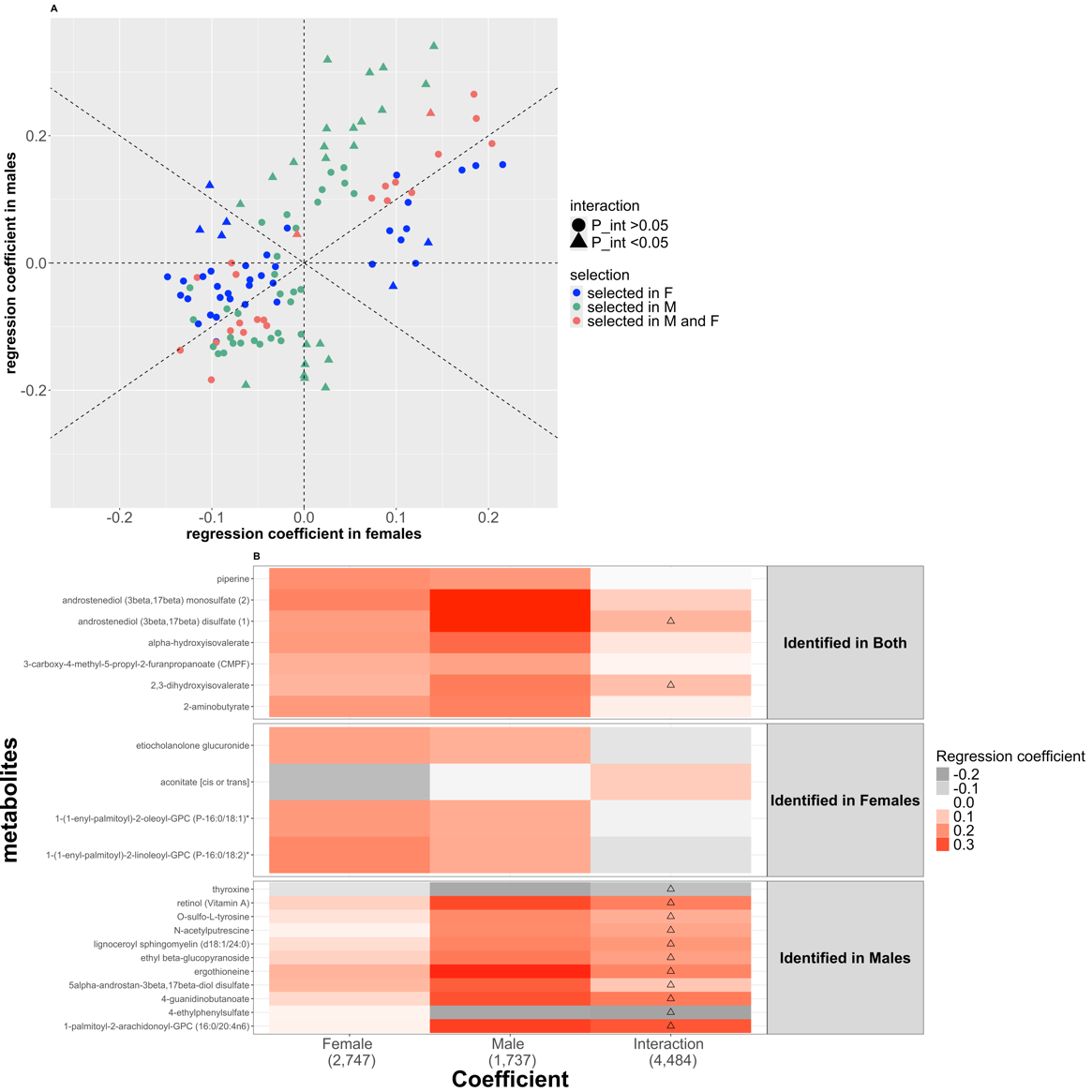
**

**Supplemental Figure 2**: **A**: The estimated regression coefficients of current alcohol consumption for metabolites selected by Elastic Net between females and males. The covariates in the regression include age, study center, birth nationality, smoking status, income, education, Alternate Healthy Eating Index 2010 (AHEI-2010), Hispanic/Latino background, and metabolomic profiling batch in both sexes. In females, menopause status, ever use of birth control medication, and hormone replacement therapy were additionally adjusted. Blue: metabolites uniquely selected in females’ model. Green: metabolites uniquely selected in males’ model. Red: metabolites selected both sexes. Δ denotes metabolites that showed a substantial difference in regression coefficients between sexes (P-interaction < 0.05). **B**: The estimated regression coefficients of most significant metabolites (FDR < 0.05) among those uniquely selected in females (4), males (11) and both sexes (7). Δ denotes metabolites that showed a substantial difference in regression coefficients between sexes (P-interaction < 0.05)


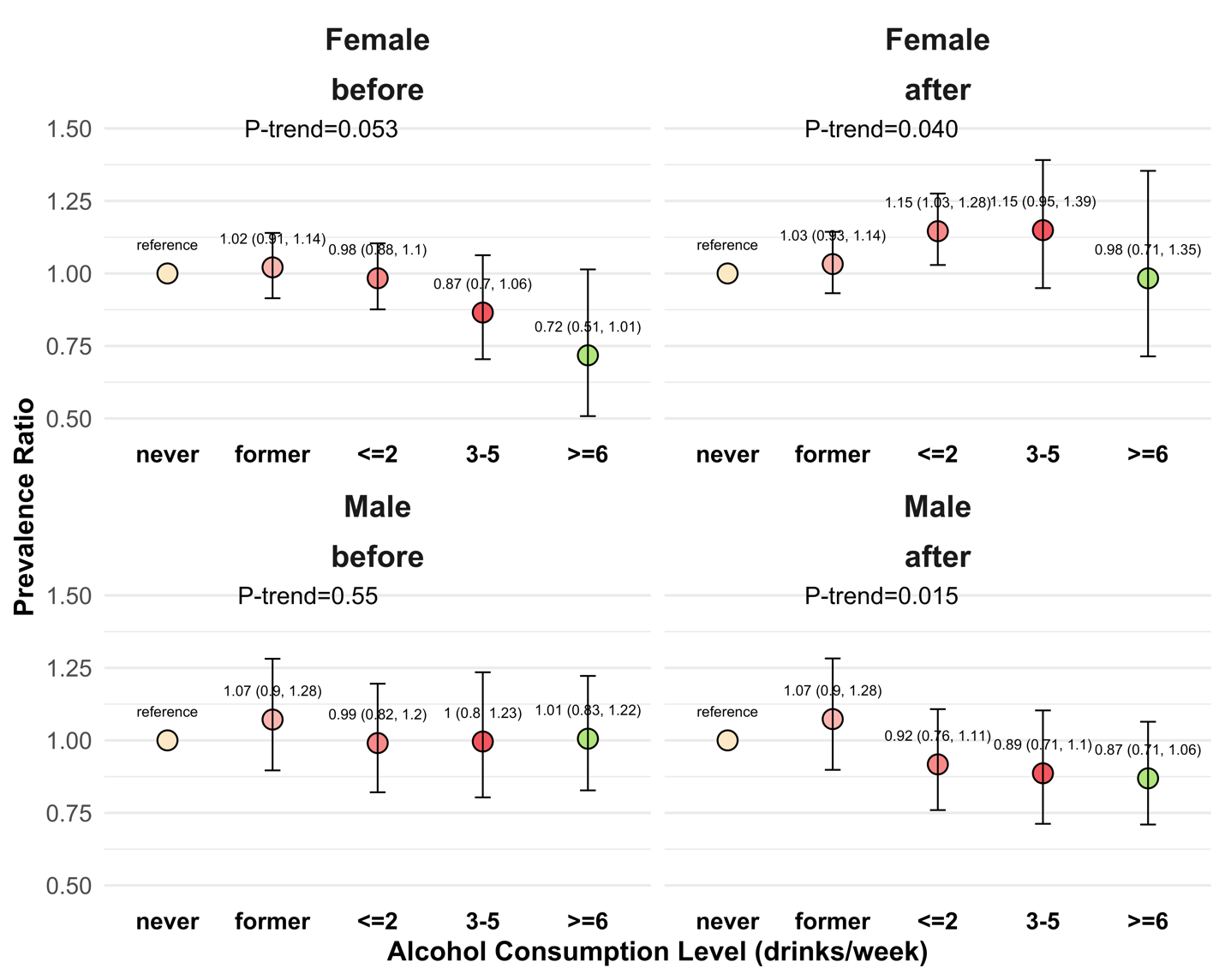


**Supplemental Figure 3:** Cross-sectional associations between alcohol consumption levels and insulin resistance before and after adjusting for sex-specific alcohol-associated metabolomic signatures in females (n=2,747) and males (n=1,737) at baseline. The prevalence ratio (PR) and 95%CI were obtained by Poisson regression and robust variance, adjusting for covariates including age, study center, birth nationality, smoking status, income, education, Alternate Healthy Eating Index 2010 (AHEI-2010), Hispanic/Latino background, and metabolomic profiling batch in both sexes and additional menopause status, ever use of birth control medication, and hormone replacement therapy in females. The P-trend was obtained by treating alcohol consumption levels as an ordinal variable, assigned values of 0 (never, reference), 1 (former), 2 ($\leq2$ drinks), 3 (3-5), and 4 (6-7 for females and 5-14 for males).

**
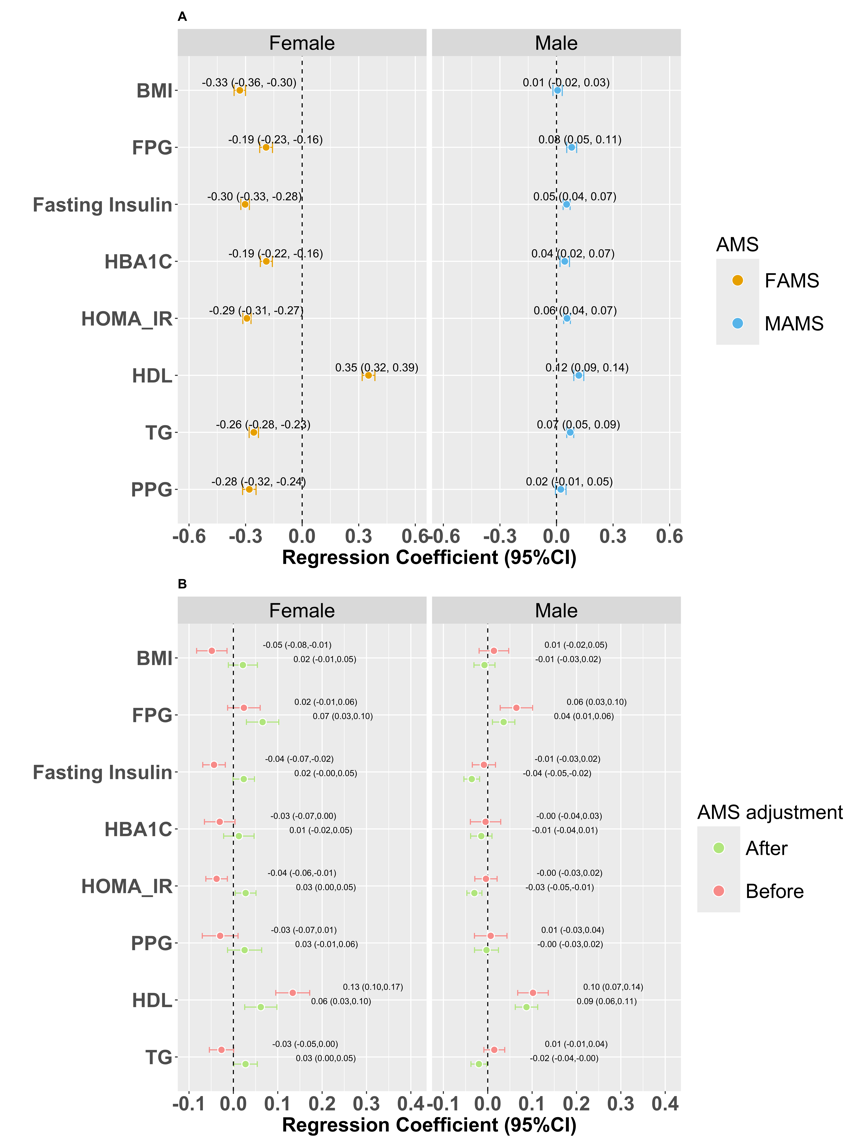
**

**Supplemental Figure 4: A**: Cross-sectional associations of sex-specific alcohol-associated metabolomic signatures (*FAMS* for females and *MAMS* for males) with glycemic traits in females (n=2,747) and males (n=1,737) at baseline. All glycemic traits were standardized (mean=0, and standard deviation =1). The regression coefficient for associations between *AMS* levels and glycemic traits were estimated by robust linear regression, treating AMS quartiles as an ordinal variable assigned values of 0 (Q1, reference), 1 (Q2), 2 (Q3), and 3 (Q4) and adjusting for covariates including age, study center, birth nationality, smoking status, income, education, Alternate Healthy Eating Index 2010 (AHEI-2010), Hispanic/Latino background, and metabolomic profiling batch in both sexes, and additional menopause status, ever use of birth control medication, and hormone replacement therapy in females. **B**: Cross-sectional associations of alcohol consumption levels with glycemic traits in females (n=2,747) and males (n=1,737) at baseline. The regression coefficients were compared between robust linear regression models before and after adjustment for the corresponding sex-specific AMS, adjusting for covariates mentioned above. All glycemic traits were standardized (mean=0, and standard deviation=1). Alcohol consumption levels were treated as an ordinal variable assigned values of 0 (never, reference), 1 (former), 2 ($\leq2$ drinks), 3 (3-5), and 4 (5-6 for females and 5-14 for males) and AMS quartiles were treated as an ordinal variable assigned values of 0 (Q1, reference), 1 (Q2), 2 (Q3), and 3 (Q4). AHEI-2010 is the alternative healthy eating index-2010. HOMA_IR is the homeostatic model assessment of insulin resistance. FPG is the fasting plasma glucose. PPG is the post 2-hour glucose. HDL is high-density lipoprotein. TG is triglycerides.


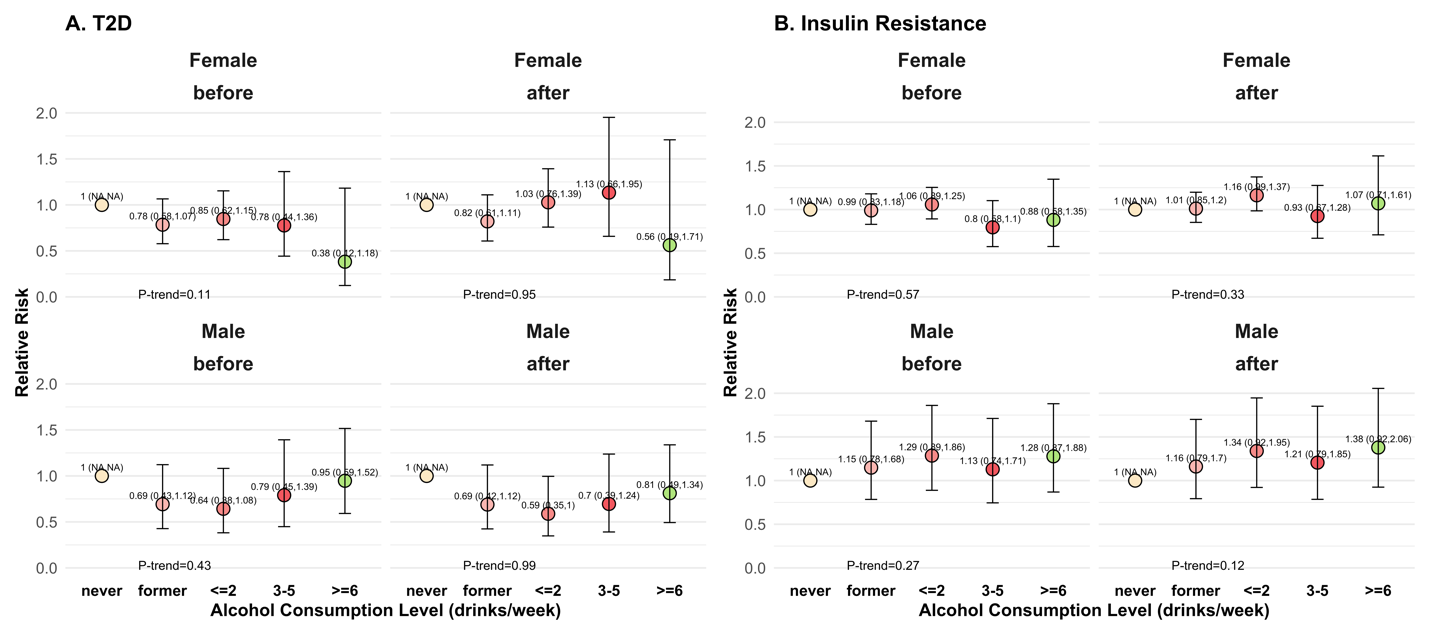


**Supplemental Figure 5**: **A:** Prospective associations between alcohol consumption levels at baseline and incident diabetes at V2 in females (n= 2,265) and males (n=1,290). **B:** Prospective associations between alcohol consumption levels at baseline and incident IR at V2 in females (n= 978) and males (n=596). The log-relative risk (RR) estimated by Poisson regression models was compared between models before and after adjustment for the corresponding sex-specific AMS, adjusting for age, study center, birth nationality, smoking status, income, education, Alternate Healthy Eating Index 2010 (AHEI-2010), Hispanic/Latino background, and metabolomic profiling batch in both sexes, and additional menopause status, ever use of birth control medication, and hormone replacement therapy in females. The P-trend was obtained by treating alcohol consumption levels as an ordinal variable assigned values of 0 (never, reference), 1 (former), 2 ($\leq2$ drinks), 3 (3-5), and 4 (5-6 for females and 5-14 for males)


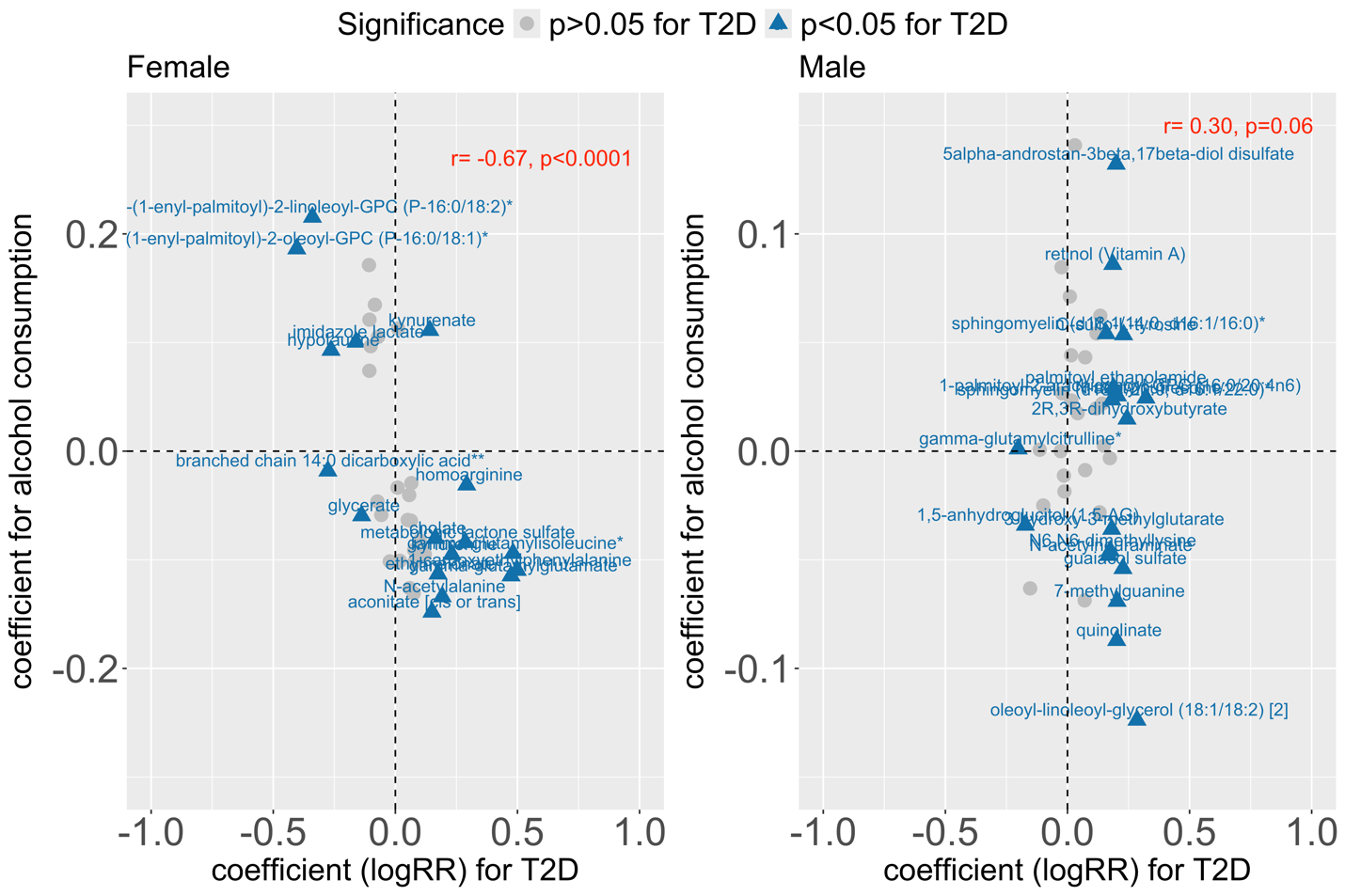


**Supplemental Figure 6**: The relationship between the changes due to alcohol consumption and effects on incident type 2 diabetes (T2D) for metabolites included in FAMS in females (2,265), and MAMS in males (1,290). The regression coefficients for alcohol consumption level were estimated by linear regression model adjusted for age, study center, birth nationality, smoking status, income, education, Alternate Healthy Eating Index 2010 (AHEI-2010), Hispanic/Latino background, and metabolomic profiling batch in both sexes, and additional menopause status, ever use of birth control medication, and hormone replacement therapy in females. The relative risk (RR) for incident diabetes was estimated by a Poisson regression model adjusted for covariates at the same set of covariates. Δ denotes metabolites that showed a significant association with incident T2D (P<0.05).

**
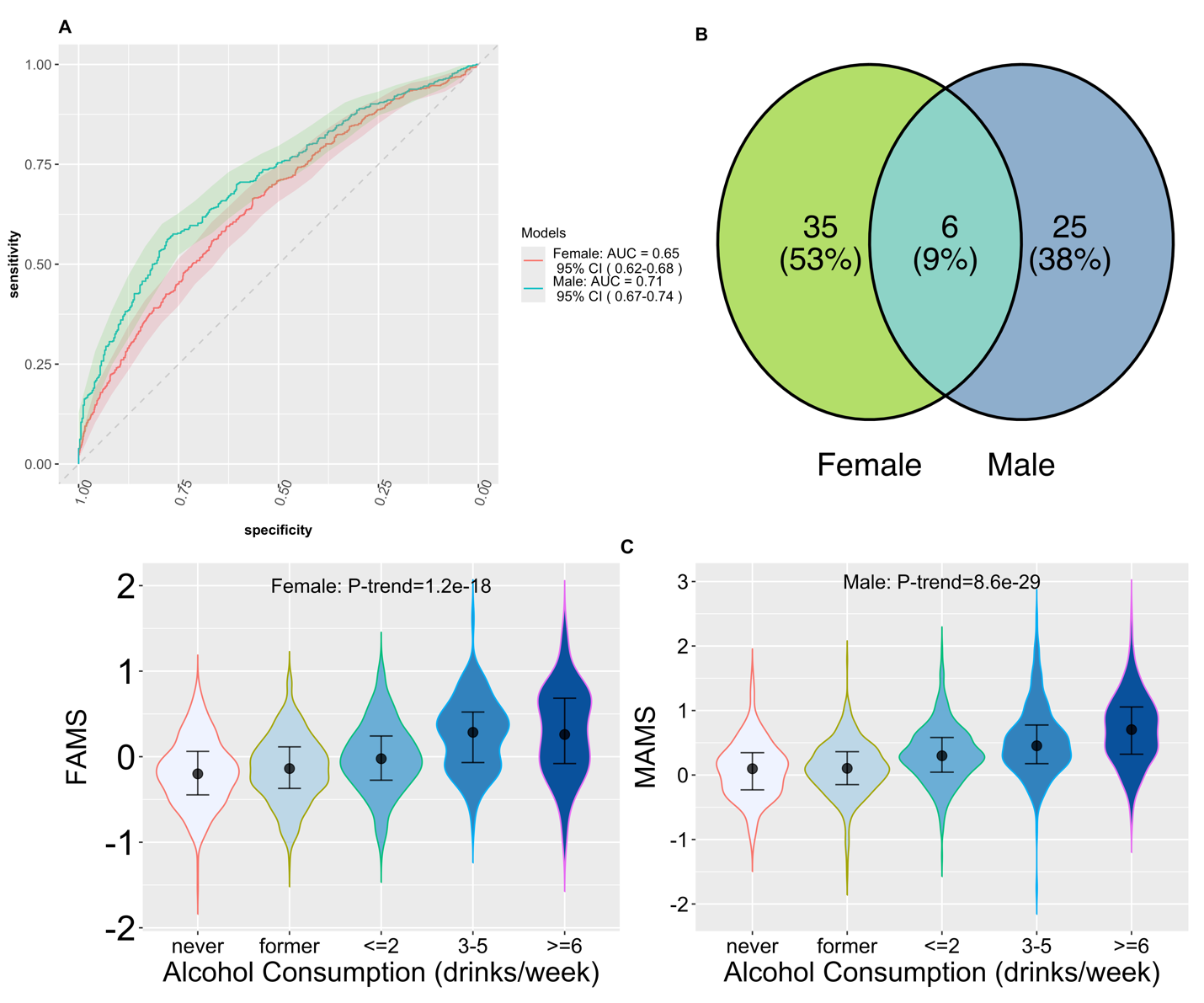
**

**Supplement Figure 7: A**: Receiver operating characteristic (ROC) curves of Elastic Net models for current alcohol consumption in the testing data sets (n=1,373 for females, red; n=869 for males, green). The 95% confidence interval of ROC (shaded area) was computed with 2000 stratified bootstrap. Elastic Net models of current alcohol consumption were constructed by ten-fold cross-validation in the training datasets (n=1,374 for females, n=868 for males). The tuning parameter was selected by minimum criteria. AUC is defined by area under curve. **B**: Venn diagram of identified metabolites by Elastic Net models for females and males. Green: metabolites uniquely identified in females; Purple: metabolites uniquely identified in males; blue: metabolites identified in both sexes. **C**: Violin plot to compare sex-specific AMSs among various alcohol consumption levels in the testing datasets in females (*FAMS*) and males (*MAMS*). The P-trend was obtained by linear regression, in which alcohol consumption levels were treated as an ordinal variable assigned values of 0 (never, reference), 1 (former), 2 ($\leq2$ drinks), 3 (3-5), and 4 (6-7 for females and 6-14 for males), adjusting for covariates including age, study center, birth nationality, smoking status, income, education, Alternate Healthy Eating Index 2010 (AHEI-2010), Hispanic/Latino background, and metabolomic profiling batch in both sexes, and additional menopause status, ever use of birth control medication, and hormone replacement therapy in females.

**
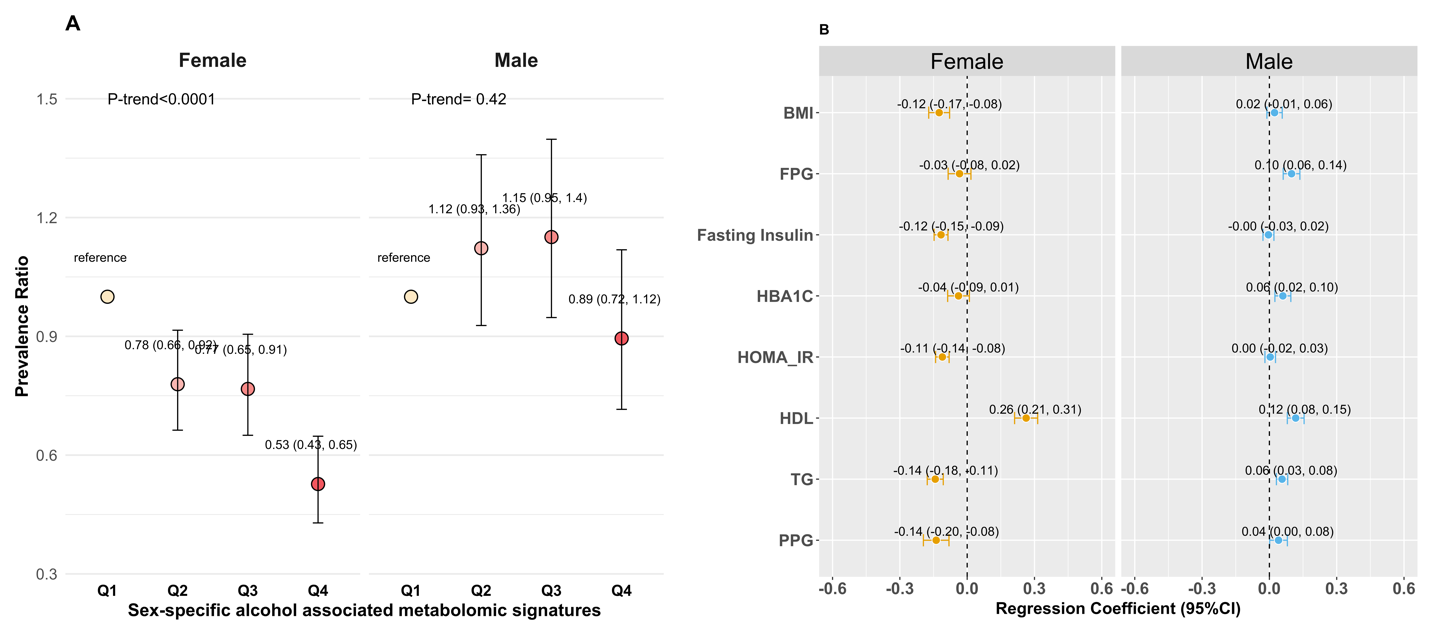
**

**Supplement Figure 8**: Cross-sectional associations of sex-specific alcohol-associated metabolomic signatures (AMSs) with insulin resistance and metabolic traits at baseline in the testing datasets for females and males. **A**: Insulin resistance. Females and males were classified into four AMS levels based on sex-specific quartiles [Q1 (reference), Q2, Q3, Q4]. Prevalence ratios (PRs) and 95% confidence intervals (CIs) were estimated using Poisson regression with robust variance, adjusted for age, study center, birth nationality, smoking status, income, education, Alternate Healthy Eating Index 2010 (AHEI-2010), Hispanic/Latino background, and metabolomic profiling batch in both sexes. In females, menopause status, ever use of birth control medication, and hormone replacement therapy were additionally adjusted. The P-trend was calculated in Poisson regression by treating sex-specific AMS quartiles as an ordinal variable assigned values of 0 (Q1, reference), 1 (Q2), 2 (Q3), and 3 (Q4). **B**: Metabolic traits. All glycemic traits were standardized (mean=0, and standard deviation =1). The regression coefficient for associations between *AMS* levels and glycemic traits were estimated by robust linear regression, treating AMS quartiles as an ordinal variable assigned values of 0 (Q1, reference), 1 (Q2), 2 (Q3), and 3 (Q4) and adjusting for covariates mentioned above. AHEI-2010 is the alternative healthy eating index-2010. HOMA-IR is the homeostatic model assessment of insulin resistance. FPG is the fasting plasma glucose. PPG is the post 2-hour glucose. HDL is high-density lipoprotein. TG is triglycerides.


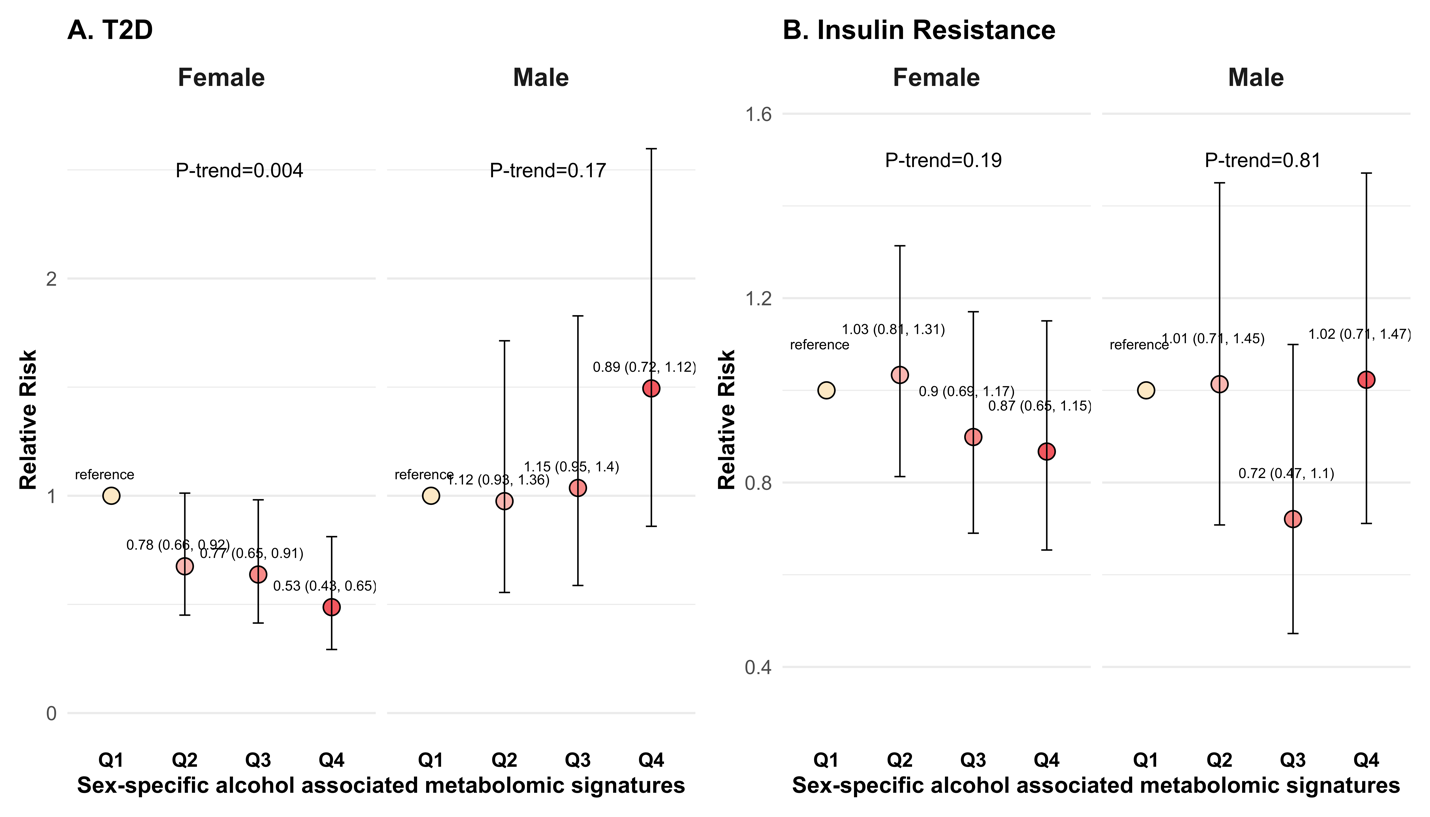


**Supplemental Figure 9:** Prospective association between sex-specific alcohol-associated metabolomic signatures (AMSs) and risk of type 2 diabetes and insulin resistance in the testing dataset for females and males. **A**: Prospective associations between sex-specific AMS levels [Q1 (reference), Q2, Q3, and Q4] at baseline and incident type 2 diabetes at V2 in females and males. Relative risks (RRs) and 95% confidence intervals (CIs) were estimated using Poisson regression, adjusted for age, study center, birth nationality, smoking status, income, education, Alternate Healthy Eating Index 2010 (AHEI-2010), Hispanic/Latino background, and metabolomic profiling batch in both sexes. In females, menopause status, ever use of birth control medication, and hormone replacement therapy were additionally adjusted. The PR-trend and P-trend were calculated in Poisson regression by treating sex-specific AMS quartiles as an ordinal variable assigned values of 0 (Q1, reference), 1 (Q2), 2 (Q3), and 3 (Q4). **B**: Prospective associations between sex-specific AMS levels [Q1 (reference), Q2, Q3, Q4] at baseline and incident insulin resistance (IR) at V2 in females and males. RRs and 95% CIs were estimated using Poisson regression, adjusted for covariates mentioned above. PR-trend and P-trend were calculated in Poisson regression by treating sex-specific AMS quartiles as an ordinal variable assigned values of 0 (Q1, reference), 1 (Q2), 2 (Q3), and 3 (Q4).

**Supplemental Tables**

| **Supplemental Table 1: Covariates by sex and alcohol consumption levels of 4,484 participants at baseline** | | | | | | | | | | |
| --- | --- | --- | --- | --- | --- | --- | --- | --- | --- | --- |
|  | Female | | | | | Male | | | | |
| Covariates | Never | Former | $\leq2$ | 3-5 | 6-7 | Never | Former | $\leq2$ | 3-5 | 6-14 |
| Age group(%) |  |  |  |  |  |  |  |  |  |  |
| 18-29 | 49 (6.5) | 67 (7.5) | 72 (8.7) | 27 (14.1) | 6 (7.5) | 33 (18.6) | 49 (9.3) | 48 (11.1) | 21 (9.5) | 38 (10.1) |
| 30-39 | 67 (8.9) | 84 (9.3) | 79 (9.6) | 27 (14.1) | 13 (16.2) | 13 (7.3) | 54 (10.2) | 56 (12.9) | 37 (16.7) | 54 (14.3) |
| 40-49 | 111 (14.8) | 163 (18.1) | 127 (15.4) | 38 (19.9) | 14 (17.5) | 22 (12.4) | 88 (16.7) | 77 (17.7) | 41 (18.6) | 69 (18.3) |
| 50-59 | 257 (34.2) | 329 (36.6) | 338 (41) | 67 (35.1) | 31 (38.8) | 48 (27.1) | 187 (35.4) | 158 (36.4) | 59 (26.7) | 133 (35.3) |
| 60-69 | 204 (27.1) | 199 (22.1) | 171 (20.7) | 24 (12.6) | 15 (18.8) | 49 (27.7) | 111 (21) | 72 (16.6) | 49 (22.2) | 61 (16.2) |
| 71-74 | 64 (8.5) | 57 (6.3) | 38 (4.6) | 8 (4.2) | 1 (1.2) | 12 (6.8) | 39 (7.4) | 23 (5.3) | 14 (6.3) | 22 (5.8) |
| US Born (%) | 44 (5.9) | 142 (15.8) | 165 (20) | 54 (28.3) | 22 (27.5) | 22 (12.4) | 93 (17.6) | 93 (21.4) | 45 (20.4) | 76 (20.2) |
| Current Smoker (%) | 79 (10.5) | 120 (13.4) | 148 (17.9) | 51 (26.7) | 31 (38.8) | 26 (14.8) | 123 (23.3) | 93 (21.5) | 68 (30.8) | 147 (39.1) |
| Income (%) |  |  |  |  |  |  |  |  |  |  |
| < $30,000 | 535 (77.4) | 633 (74.7) | 509 (65.8) | 107 (58.2) | 56 (70.9) | 110 (66.7) | 343 (68.5) | 232 (55.1) | 116 (54) | 206 (56.1) |
| $30,000 or more | 156 (22.6) | 214 (25.3) | 265 (34.2) | 77 (41.8) | 23 (29.1) | 55 (33.3) | 158 (31.5) | 189 (44.9) | 99 (46) | 161 (43.9) |
| Education(%) |  |  |  |  |  |  |  |  |  |  |
| Less than High School | 260 (34.7) | 361 (40.2) | 231 (28) | 49 (25.7) | 27 (33.8) | 52 (29.4) | 206 (39.1) | 117 (27) | 69 (31.2) | 131 (34.8) |
| High School or Equivalent | 184 (24.6) | 231 (25.7) | 197 (23.9) | 44 (23) | 21 (26.2) | 50 (28.2) | 148 (28.1) | 120 (27.6) | 57 (25.8) | 113 (30.1) |
| Greater than High School or Equivalent | 305 (40.7) | 307 (34.1) | 396 (48.1) | 98 (51.3) | 32 (40) | 75 (42.4) | 173 (32.8) | 197 (45.4) | 95 (43) | 132 (35.1) |
| Center (%) |  |  |  |  |  |  |  |  |  |  |
| Bronx | 124 (16.5) | 294 (32.7) | 228 (27.6) | 62 (32.5) | 33 (41.2) | 21 (11.9) | 139 (26.3) | 112 (25.8) | 47 (21.3) | 91 (24.1) |
| Chicago | 161 (21.4) | 210 (23.4) | 191 (23.2) | 28 (14.7) | 11 (13.8) | 34 (19.2) | 134 (25.4) | 114 (26.3) | 61 (27.6) | 104 (27.6) |
| Miami | 377 (50.1) | 155 (17.2) | 188 (22.8) | 47 (24.6) | 20 (25) | 111 (62.7) | 130 (24.6) | 111 (25.6) | 63 (28.5) | 113 (30) |
| San Diego | 90 (12) | 240 (26.7) | 218 (26.4) | 54 (28.3) | 16 (20) | 11 (6.2) | 125 (23.7) | 97 (22.4) | 50 (22.6) | 69 (18.3) |
| Hispanic Background (%) |  |  |  |  |  |  |  |  |  |  |
| Dominican | 63 (8.4) | 126 (14) | 108 (13.1) | 21 (11) | 16 (20) | 7 (4) | 50 (9.5) | 46 (10.6) | 18 (8.1) | 36 (9.6) |
| Central American | 141 (18.8) | 77 (8.6) | 64 (7.8) | 16 (8.4) | 8 (10) | 24 (13.6) | 59 (11.2) | 29 (6.7) | 13 (5.9) | 46 (12.3) |
| Cuban | 212 (28.3) | 84 (9.3) | 120 (14.6) | 26 (13.6) | 11 (13.8) | 81 (45.8) | 78 (14.8) | 74 (17.1) | 50 (22.6) | 75 (20) |
| Mexican | 192 (25.6) | 363 (40.4) | 307 (37.3) | 65 (34) | 21 (26.2) | 24 (13.6) | 171 (32.4) | 155 (35.7) | 79 (35.7) | 129 (34.4) |
| Puerto Rican | 63 (8.4) | 162 (18) | 135 (16.4) | 40 (20.9) | 18 (22.5) | 21 (11.9) | 111 (21.1) | 80 (18.4) | 39 (17.6) | 57 (15.2) |
| South American | 63 (8.4) | 63 (7) | 64 (7.8) | 16 (8.4) | 3 (3.8) | 16 (9) | 45 (8.5) | 37 (8.5) | 15 (6.8) | 20 (5.3) |
| Other | 16 (2.1) | 24 (2.6) | 25 (3.0) | 7 (3.7) | 3 (3.8) | 4 (2.2) | 13 (2.5) | 13 (3.0) | 7 (3.2) | 12 (3.2) |
| Menopause (%) |  |  |  |  |  |  |  |  |  |  |
| No | 320 (49.7) | 439 (58.5) | 417 (59.5) | 111 (70.3) | 47 (68.1) |  |  |  |  |  |
| Yes | 324 (50.3) | 311 (41.5) | 284 (40.5) | 47 (29.7) | 22 (31.9) |  |  |  |  |  |
| Ever use birth control medication (%) |  |  |  |  |  |  |  |  |  |  |
| No | 293 (43.5) | 307 (38.2) | 248 (33.7) | 56 (33.9) | 26 (36.1) |  |  |  |  |  |
| Yes | 380 (56.5) | 496 (61.8) | 487 (66.3) | 109 (66.1) | 46 (63.9) |  |  |  |  |  |
| Hormone Replacement Therapy (%) |  |  |  |  |  |  |  |  |  |  |
| No | 716 (97) | 857 (96.6) | 772 (95.3) | 180 (96.8) | 71 (94.7) |  |  |  |  |  |
| Yes | 22 (3) | 30 (3.4) | 38 (4.7) | 6 (3.2) | 4 (5.3) |  |  |  |  |  |
| AHEI2010 | 46.9 (42.6, 52.3) | 48 (42.7, 53.2) | 47.8 (42.3, 52.8) | 45.7 (40.5, 52.1) | 46.7 (41.3, 50.6) | 46.1 (42.7, 50.5) | 48.9 (43.7, 54.9) | 49.3 (44.2, 55.9) | 49.3 (44, 54.4) | 49.4 (44.4, 55) |
| IR (%) | 343 (45.6) | 406 (45.4) | 348 (42.2) | 70 (36.6) | 24 (30) | 85 (48) | 260 (49.2) | 192 (44.3) | 98 (44.3) | 165 (43.9) |
| BMI (kg/m2) | 28.7 (25.7, 32.8) | 29.7 (26, 33.7) | 28.3 (25.4, 32.8) | 27.5 (24.2, 31.6) | 27.6 (25.5, 32.7) | 27.5 (23.8, 30.6) | 28.6 (25.5, 31.5) | 28.2 (25.4, 31) | 27.8 (25.4, 30.6) | 28.2 (25.3, 31.6) |
| Fasting glucose (mg/dL) | 92 (87, 97) | 92 (87, 98) | 92 (87, 97) | 91 (86, 96.2) | 91 (88, 97.2) | 94 (89, 100) | 96 (90, 101) | 96 (90, 101) | 96 (91, 101.5) | 96 (91, 102) |
| HbA1c (mmol mol-1) | 37 (34, 39) | 37 (34, 40) | 37 (34, 40) | 36 (33, 38) | 37 (32, 39) | 36 (33, 39) | 37 (34, 40) | 37 (34, 39) | 37 (34, 39) | 37 (33, 39) |
| Fasting insulin (mIU/L) | 10.2 (7.2, 15.2) | 10.2 (7, 15.6) | 9.7 (6.6, 14.6) | 8.6 (5.9, 13.3) | 7.6 (5.9, 11.6) | 10.5 (6, 16) | 10.3 (6, 16) | 9.6 (6, 15.5) | 9 (6.6, 13.6) | 9.6 (5.9, 14.1) |
| HOMA-IR | 2.3 (1.6, 3.6) | 2.4 (1.6, 3.6) | 2.2 (1.4, 3.4) | 2 (1.3, 3.1) | 1.7 (1.3, 2.8) | 2.4 (1.4, 3.8) | 2.5 (1.4, 3.9) | 2.3 (1.4, 3.7) | 2.2 (1.5, 3.4) | 2.3 (1.4, 3.5) |
| PPG (mg/dL) | 120 (100, 144) | 120 (101, 143) | 116 (98, 137) | 109.5 (90, 127.8) | 122 (100.5, 134) | 117 (94, 143) | 110 (86, 132) | 107 (88.2, 130) | 106 (86, 129) | 109 (89.2, 133) |
| HDL (mg/dL) | 51 (44, 60) | 51 (44, 58.5) | 53 (45, 62) | 56 (49, 66) | 57 (50.8, 68.2) | 42 (37, 48) | 42 (36, 50) | 44 (38, 52) | 45 (39, 51) | 46 (39, 54) |
| TG (mg/dL) | 105 (78, 150) | 110 (77, 152) | 100 (73, 139) | 90 (72, 135) | 100 (78.8, 135) | 128 (87, 190.5) | 125 (80, 188) | 114 (79, 170.8) | 120 (85.8, 162) | 137 (85.1, 199) |

Values are denoted as median (IQR1, IQR3). AHEI-2010 is the alternative healthy eating index-2010. HOMA-IR is the homeostatic model assessment of insulin resistance. PPG is the post 2-hour glucose. HDL is high-density lipoprotein. TG is triglycerides.

| Supplemental Table 2: Associations of Current Alcohol Consumption and Its Interaction with Sex on Uniquely Selected Metabolites Identified by Elastic Net in Female and Male. | | | | | |
| --- | --- | --- | --- | --- | --- |
| Chemical Name | Estimate | P-value | FDR | Alcohol-sex interaction p-value | Sex selected in |
| kynurenate | 0.11119867 | 0.00474498 | 1 | 0.54253148 | Female |
| cholate | -0.0802757 | 0.04046983 | 1 | 0.50761289 | Female |
| fumarate | -0.089344 | 0.01980056 | 1 | **0.02451225** | Female |
| gamma-glutamylglutamate | -0.1147245 | 0.00065455 | 0.38029501 | 0.1700216 | Female |
| gluconate | -0.0952268 | 0.01053998 | 1 | 0.66810981 | Female |
| cortisol | 0.11288987 | 0.00483694 | 1 | 0.92539996 | Female |
| hypotaurine | 0.0929174 | 0.01258956 | 1 | 0.10654946 | Female |
| malate | -0.1023396 | 0.01001016 | 1 | **0.00068725** | Female |
| nonadecanoate (19:0) | -0.0463177 | 0.24434048 | 1 | 0.55645478 | Female |
| dihydroorotate | -0.0639581 | 0.1021118 | 1 | 0.87663751 | Female |
| glycerate | -0.0593815 | 0.0920018 | 1 | 0.45976766 | Female |
| N-acetylalanine | -0.1339624 | 0.00038116 | 0.22336199 | 0.22204446 | Female |
| ethylmalonate | -0.1130031 | 0.00387432 | 1 | **0.013773** | Female |
| imidazole lactate | 0.10043397 | 0.00748492 | 1 | 0.82586651 | Female |
| kynurenine | -0.0951093 | 0.01647876 | 1 | 0.63135497 | Female |
| quinate | 0.09664327 | 0.0051877 | 1 | **0.01487962** | Female |
| homoarginine | -0.0310618 | 0.41655954 | 1 | 0.82220154 | Female |
| indoleacetate | -0.0403752 | 0.32214675 | 1 | 0.27715354 | Female |
| aconitate [cis or trans] | -0.1481153 | 7.67E-05 | 0.04557857 | 0.10920671 | Female |
| gamma-glutamylisoleucine* | -0.0940106 | 0.00955526 | 1 | 0.20074465 | Female |
| glutarylcarnitine (C5-DC) | 0.10516708 | 0.00473417 | 1 | 0.22935724 | Female |
| o-cresol sulfate | -0.0294159 | 0.32995789 | 1 | 0.38660887 | Female |
| gamma-CEHC | -0.1014951 | 0.00917496 | 1 | 0.89234725 | Female |
| eicosanedioate (C20-DC) | 0.13474591 | 0.00020231 | 0.11976988 | **0.04454345** | Female |
| 2-piperidinone | -0.0633482 | 0.11720942 | 1 | 0.20608444 | Female |
| acisoga | -0.0822973 | 0.02888438 | 1 | 0.52101111 | Female |
| etiocholanolone glucuronide | 0.17130619 | 1.94E-06 | 0.00116698 | 0.30965809 | Female |
| 3-hydroxypyridine sulfate | 0.07402199 | 0.03101859 | 1 | 0.24215172 | Female |
| 1-(1-enyl-palmitoyl)-2-oleoyl-GPC (P-16:0/18:1)* | 0.18631035 | 3.60E-06 | 0.00216286 | 0.64874594 | Female |
| 1-(1-enyl-palmitoyl)-2-linoleoyl-GPC (P-16:0/18:2)* | 0.21545079 | 3.19E-08 | 1.93E-05 | 0.2889604 | Female |
| hexadecadienoate (16:2n6) | -0.1009231 | 0.0065442 | 1 | 0.11332402 | Female |
| linoleoyl-linoleoyl-glycerol (18:2/18:2) [1]* | -0.1308057 | 0.00064623 | 0.37610647 | 0.30225664 | Female |
| (N(1) + N(8))-acetylspermidine | -0.0909919 | 0.01796471 | 1 | 0.50352489 | Female |
| glutamine conjugate of C6H10O2 (1)* | -0.1260898 | 0.00146674 | 0.83164269 | 0.5927315 | Female |
| 1-carboxyethylphenylalanine | -0.1096766 | 0.00433695 | 1 | 0.08701462 | Female |
| N-acetyl-isoputreanine | -0.0586921 | 0.13024739 | 1 | 0.72127092 | Female |
| eicosenedioate (C20:1-DC)* | 0.12105779 | 0.00113501 | 0.64695696 | 0.0659105 | Female |
| 3,5-dichloro-2,6-dihydroxybenzoic acid | -0.0335683 | 0.36991925 | 1 | 0.28476066 | Female |
| metabolonic lactone sulfate | -0.0840647 | 0.03721981 | 1 | **0.00734861** | Female |
| branched chain 14:0 dicarboxylic acid** | -0.0182036 | 0.6229815 | 1 | 0.06167017 | Female |
| 3-hydroxy-3-methylglutarate | -0.1183052 | 0.01101366 | 1 | 0.12312947 | Male |
| quinolinate | -0.1414002 | 0.00387244 | 1 | 0.42717719 | Male |
| N-acetylputrescine | 0.21111098 | 2.02E-05 | 0.01176491 | **0.00631013** | Male |
| citrulline | -0.1222463 | 0.00577474 | 1 | 0.16907694 | Male |
| ornithine | -0.1120135 | 0.00882311 | 1 | 0.18582592 | Male |
| retinol (Vitamin A) | 0.30694213 | 9.99E-11 | 6.01E-08 | **8.08E-05** | Male |
| adenine | -0.0416848 | 0.38012341 | 1 | 0.48906169 | Male |
| caprylate (8:0) | 0.12546114 | 0.00943214 | 1 | 0.25617422 | Male |
| pentadecanoate (15:0) | -0.079296 | 0.08260966 | 1 | 0.64139704 | Male |
| N-acetylleucine | -0.0455043 | 0.34742203 | 1 | 0.72087169 | Male |
| thyroxine | -0.1919174 | 5.93E-05 | 0.03429516 | **0.02061972** | Male |
| 3-aminoisobutyrate | 0.07597713 | 0.1292582 | 1 | 0.14924148 | Male |
| 4-hydroxyphenylpyruvate | -0.0721586 | 0.13539131 | 1 | 0.59970324 | Male |
| N-acetylneuraminate | -0.1276768 | 0.00550671 | 1 | 0.09156687 | Male |
| gamma-glutamylhistidine | 0.05467943 | 0.25135231 | 1 | 0.0914215 | Male |
| palmitoyl ethanolamide | 0.14243313 | 0.00479994 | 1 | 0.07305503 | Male |
| phenylacetate | -0.1810221 | 0.00017815 | 0.10100963 | **0.00686901** | Male |
| 5-hydroxylysine | -0.1173089 | 0.02085285 | 1 | 0.74045939 | Male |
| 4-guanidinobutanoate | 0.2990942 | 2.03E-11 | 1.23E-08 | **3.24E-05** | Male |
| 1,5-anhydroglucitol (1,5-AG) | 0.13469903 | 0.00576479 | 1 | **0.00135017** | Male |
| tartronate (hydroxymalonate) | -0.1270899 | 0.00514081 | 1 | **0.02530352** | Male |
| pro-hydroxy-pro | -0.1258445 | 0.00457393 | 1 | 0.73457265 | Male |
| erythronate* | -0.0891101 | 0.05253704 | 1 | 0.2610994 | Male |
| phenylacetylglutamine | -0.1766622 | 0.00040664 | 0.22690693 | **0.00857262** | Male |
| 7-methylguanine | 0.09232573 | 0.05200677 | 1 | **0.01756781** | Male |
| phenol sulfate | -0.0486235 | 0.31878538 | 1 | 0.93831434 | Male |
| octadecanedioate (C18-DC) | 0.11527601 | 0.02427526 | 1 | 0.15107912 | Male |
| 4-ethylphenylsulfate | -0.1960005 | 9.47E-06 | 0.00554385 | **0.00065972** | Male |
| 5alpha-androstan-3beta,17beta-diol disulfate | 0.28068436 | 3.59E-12 | 2.17E-09 | **0.03808762** | Male |
| taurocholenate sulfate* | 0.01043525 | 0.83134397 | 1 | 0.73772469 | Male |
| N-acetyl-beta-alanine | 0.10907397 | 0.03604525 | 1 | 0.4056264 | Male |
| 16a-hydroxy DHEA 3-sulfate | -0.0610568 | 0.13775098 | 1 | 0.06828541 | Male |
| ergothioneine | 0.34046521 | 3.08E-14 | 1.87E-11 | **6.56E-05** | Male |
| 2R,3R-dihydroxybutyrate | 0.09558296 | 0.03371212 | 1 | 0.46391554 | Male |
| sulfate* | -0.128116 | 0.00974276 | 1 | **0.03247509** | Male |
| S-methylcysteine | -0.0178643 | 0.71227145 | 1 | 0.97468914 | Male |
| oleoyl-linoleoyl-glycerol (18:1/18:2) [2] | -0.0389249 | 0.45089389 | 1 | 0.33889962 | Male |
| (R)-3-hydroxybutyrylcarnitine | 0.18271274 | 9.84E-05 | 0.05649934 | **0.00633199** | Male |
| guaiacol sulfate | -0.122064 | 0.01125551 | 1 | 0.30435759 | Male |
| sphingomyelin (d18:1/14:0, d16:1/16:0)* | 0.18371298 | 0.00013233 | 0.0754252 | **0.00470143** | Male |
| methionine sulfone | -0.1262156 | 0.0061183 | 1 | 0.72984151 | Male |
| O-sulfo-L-tyrosine | 0.21191037 | 7.08E-06 | 0.00415511 | **0.01303683** | Male |
| phenylacetylcarnitine | -0.1523542 | 0.00147354 | 0.7957122 | **0.0339486** | Male |
| sphingomyelin (d18:1/20:0, d16:1/22:0)* | 0.16445979 | 0.00094638 | 0.51956495 | **0.00681199** | Male |
| lignoceroyl sphingomyelin (d18:1/24:0) | 0.22183373 | 5.10E-06 | 0.00301019 | **0.00123801** | Male |
| adipoylcarnitine (C6-DC) | -0.1426149 | 0.00262583 | 1 | 0.46302745 | Male |
| 1-palmitoyl-2-arachidonoyl-GPC (16:0/20:4n6) | 0.31923039 | 6.55E-11 | 3.95E-08 | **8.78E-07** | Male |
| 3-carboxy-4-methyl-5-pentyl-2-furanpropionate (3-CMPFP)** | 0.14980301 | 0.00316396 | 1 | 0.06400461 | Male |
| hydroxyasparagine** | -0.1314887 | 0.00181778 | 0.97796355 | 0.76163175 | Male |
| gamma-glutamylcitrulline* | -0.1593563 | 0.00088561 | 0.48708577 | **0.0362202** | Male |
| ethyl beta-glucopyranoside | 0.24020997 | 3.23E-07 | 0.00019317 | **0.00417246** | Male |
| N6,N6-dimethyllysine | 0.06382408 | 0.17872093 | 1 | 0.11002969 | Male |
| dodecadienoate (12:2)* | 0.15818689 | 0.00130354 | 0.70651769 | **0.00881203** | Male |
| Fibrinopeptide B (1-13)** | -0.1102336 | 0.02023302 | 1 | 0.12107271 | Male |
| Table Note: Results were obtained from regression models adjusted for covariates as described in the methods. | | | | | |
